# The Structural Dynamics of Engineered β-Lactamases Vary Broadly on Three Timescales yet Sustain Native Function

**DOI:** 10.1038/s41598-019-42866-8

**Published:** 2019-04-30

**Authors:** Sophie M. C. Gobeil, Maximillian C. C. J. C. Ebert, Jaeok Park, Donald Gagné, Nicolas Doucet, Albert M. Berghuis, Jürgen Pleiss, Joelle N. Pelletier

**Affiliations:** 1 Département de biochimie and Center for Green Chemistry and Catalysis (CGCC), Université de Montréal, Montréal, QC H3T 1J4 Canada; 2 PROTEO, the Québec Network for Research on Protein Function, Engineering, and Applications, Québec, QC G1V 0A6 Canada; 3 Department of Biochemistry, McGill University, Montréal, QC H3G 1Y6 Canada; 4 GRASP, Groupe de Recherche Axé sur la Structure des Protéines, H3G 0B1 Montréal, Canada; 5 INRS-Institut Armand-Frappier, Université du Québec, Laval, QC H7V 1B7 Canada; 6 Institute of Biochemistry and Technical Biochemistry, University of Stuttgart, Stuttgart, 70569 Germany; 7 Département de chimie, Université de Montréal, Montréal, QC H3T 1J4 Canada

**Keywords:** X-ray crystallography, Solution-state NMR, Protein design

## Abstract

Understanding the principles of protein dynamics will help guide engineering of protein function: altering protein motions may be a barrier to success or may be an enabling tool for protein engineering. The impact of dynamics on protein function is typically reported over a fraction of the full scope of motional timescales. If motional patterns vary significantly at different timescales, then only by monitoring motions broadly will we understand the impact of protein dynamics on engineering functional proteins. Using an integrative approach combining experimental and *in silico* methodologies, we elucidate protein dynamics over the entire span of fast to slow timescales (ps to ms) for a laboratory-engineered system composed of five interrelated β-lactamases: two natural homologs and three laboratory-recombined variants. Fast (ps-ns) and intermediate (ns-µs) dynamics were mostly conserved. However, slow motions (µs-ms) were few and conserved in the natural homologs yet were numerous and widely dispersed in their recombinants. Nonetheless, modified slow dynamics were functionally tolerated. Crystallographic B-factors from high-resolution X-ray structures were partly predictive of the conserved motions but not of the new slow motions captured in our solution studies. Our inspection of protein dynamics over a continuous range of timescales vividly illustrates the complexity of dynamic impacts of protein engineering as well as the functional tolerance of an engineered enzyme system to new slow motions.

## Introduction

Dynamic structural biology describes proteins as 4-dimensional objects, where motions over time are considered as fundamental descriptors of protein structure. Protein motions occur over various timescales: rapid bond angle vibrations and torsions in the fs to ns timescale cause backbone and sidechain motions, while slower motions in the ns to ms timescale mediate loop and domain rearrangements by exploring conformational states that are separated by higher energy barriers^1,2^. Protein dynamics have been correlated to function for various proteins^3–7^. In addition, transitions between conformational states are thought to underlie functional diversity^8–11^.

If protein dynamics are functionally relevant, it is reasonable to expect that dynamics should be conserved throughout evolution, as are – to differing extents – protein sequence, structure and function. Indeed, evolutionary conservation of dynamics was observed in diverse protein folds, consistent with specific motions being linked to function^12–19^. Nonetheless, motions are not completely conserved, and there is evidence that the variation of protein dynamics contributes to the evolvability of function^12,20–24^.

It is tempting to assume that there is not only one relationship between dynamics and function, but multiple influences that are specific to distinct timescales. To this effect, we previously examined the motions of a fully functional engineered β-lactamase that had been recombined from the native class A β-lactamases TEM-1 and PSE-4^25^. We observed that fast dynamics (ps-ns timescale) in the engineered enzyme were highly conserved with the native homologs; in parallel, it exhibited new, non-native slow dynamics (µs-ms) that were broadly distributed throughout the protein and contrasted sharply with the static nature of the native homologs^17^.

While intriguing, that study examined a single engineered β-lactamase and left a knowledge gap relative to the intermediary ns-µs motions. The ns-µs motions can play an important role in β-lactamase function, such as triggering kinetic alterations that provide inhibitor resistance in a clinical variant of TEM-1 β-lactamase^26^. Nonetheless, capturing the relation between protein dynamics and function is challenging because distinct methods are required to examine each timescale.

**Figure 1:**
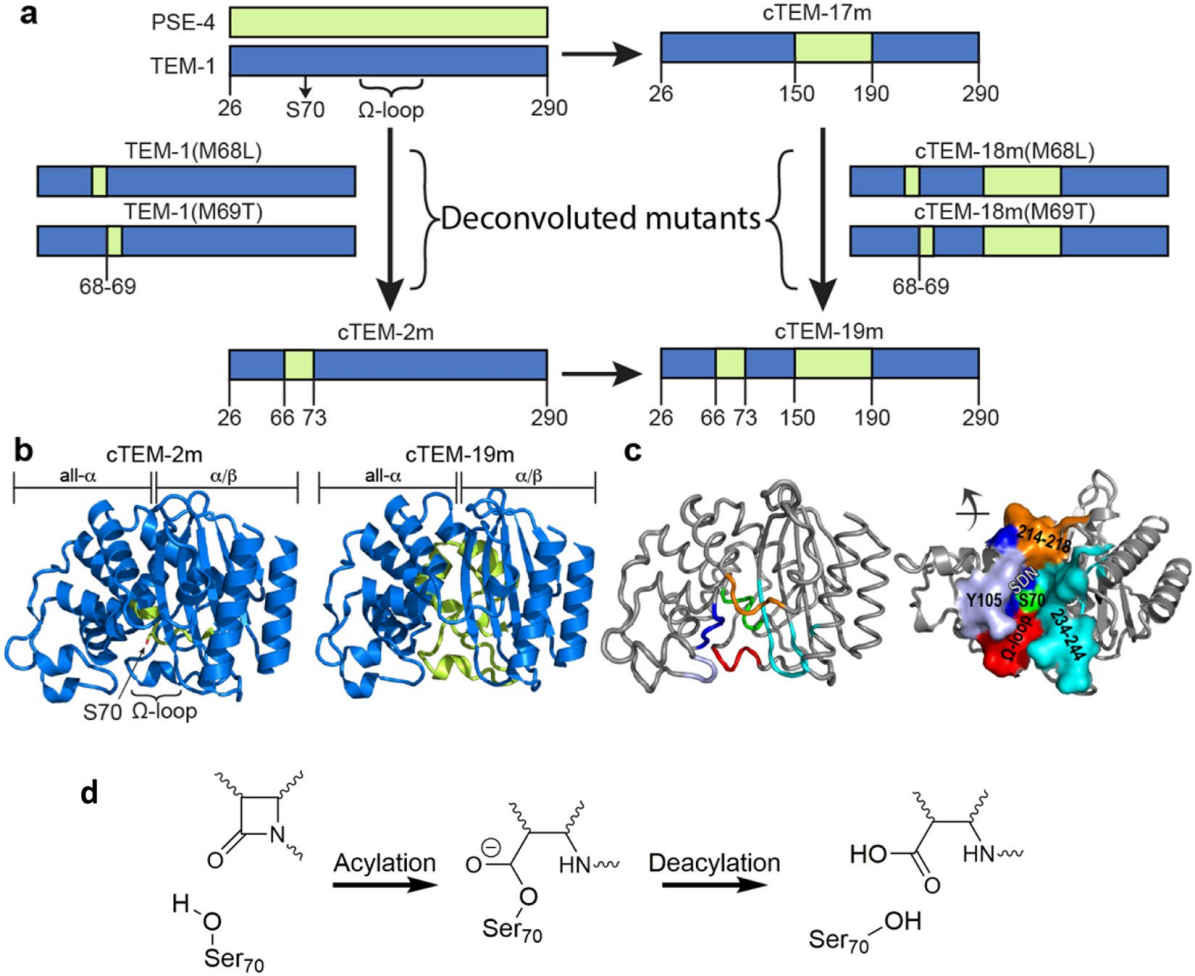
Hybrid active sites of the β-lactamase chimeras under investigation. (**a**) The native class A TEM-1 and PSE-4 β-lactamases (40% sequence identity) were recombined to yield chimeras^31,32^. Segments originating from TEM-1 (blue) and PSE-4 (green) in the chimeras cTEM-2m, cTEM-17m, cTEM-19m; ‘c’ indicates chimera and ‘m’ the number of substitutions relative to TEM-1. Deconvolution of the mutations at positions 68 and 69 gave cTEM-18m(M68L), cTEM-18m(M69T), TEM-1(M68L) and TEM-1(M69T). Numbering according to Ambler^72^. The catalytic nucleophile (Ser70) and Ω-loop are indicated. (**b**) Structural representation of cTEM-2m (PDB ID: 4MEZ) and cTEM-19m (PDB ID: 4R4S), colored as in (**a**), highlight the hybrid active site composition at the interface of the all-α and α/β domains. (**c**) Active-site walls, set in TEM-1 (PDB ID: 1XPB). Green: S70 wall (Met69-Lys73); lilac, Y105 wall (Val 103-Ser106); dark blue, SDN wall (Met129-Asn132); red, Ω-loop wall (Glu166-Asn170); orange, 214–218 wall; and cyan, 234–244 wall. (Right) Solvent-accessible surface of the active-site walls. (**d**) Reaction scheme for the hydrolysis of β-lactams by β-lactamases.

To examine a continuous spectrum of motions, here we have applied an integrative approach to determine the structural dynamics of the native class A TEM-1 and PSE-4 β-lactamases. We have elucidated their protein dynamics over timescales ranging from ps to ms by combining previously acquired results of NMR relaxation, crystallographic and steady-state kinetic data^17,27–31^ with new molecular dynamics simulations. We further expand this investigation to engineered enzyme variants by examining the same range of motions in a closely interrelated system composed of three recombined and functionally selected variants of TEM-1 and PSE-4, or ‘chimeras’ (Fig. 1). The three chimeras investigated include the above-mentioned engineered β-lactamase and two new chimeras^17^. They were specifically chosen because the recombination events were focused at the active site; all display high *in-vivo* function, allowing an investigation of the impact of active site engineering on protein motions. Importantly, two of the chimeras were recombined in different areas of the active site and the third chimera combines both modifications, thus forming a tightly related family of native and engineered enzymes^25,32^.

The three engineered chimeras displayed a remarkable conservation of dynamics on the timescale of the fast and intermediate motions examined (ps to ns and ns to µs) and were generally similar to the native TEM-1 and PSE-4. In a striking contrast, all three chimeras displayed slow dynamics (µs to ms or slower) that are not present in the rather static TEM-1 and PSE-4^17^. The new motions are broadly distributed over the entire protein; the pattern of motions differed among the chimeras according to their active-site recombinations. We thus demonstrate that a variety of non-native slow motions, including active-site motions that occur near the frequency of turnover, are compatible with β-lactamase activity in engineered variants.

Finally, we examine which of the timescales of motions examined, if any, are reflected in the B-factors of the crystal structures of these β-lactamases. High crystallographic B-factors are frequently interpreted as an expression of protein motion^22,33,34^. However, it is difficult to establish the timescale of motions captured by crystallographic B-factors, and moreover, they are affected by technical factors unrelated to dynamics^35,36^. While higher B-factor values agree broadly with the conserved fast and intermediate motions in our system, we observe no significant correlation between crystallographic B-factors and the slow timescale dynamics.

## Results

The system of five interrelated, functional β-lactamases stems from the recombination of two homologs, the TEM-1 penicillinase and the PSE-4 carbenicillinase (Fig. 1)^37^. They were previously recombined to yield chimeric β-lactamases that were selected for hydrolytic function toward ampicillin^25,32^. Chimera cTEM-2m includes residues 66–73 from PSE-4 (sequences are in Fig. S1). This introduces the substitutions Met68Leu and Met69Thr relative to TEM-1 in the S70 wall at the core of the active site; they are immediate neighbors of the catalytic nucleophile Ser70. Chimera cTEM-17m^17,29^ includes segment 150–190 from PSE-4, resulting in 17 substitutions in the catalytically-relevant Ω-loop (161–179) and its adjacent helices. The Ω-loop active-site wall contains the conserved Glu166, proposed to serve as a general base in the catalytic acylation and deacylation steps (Fig. 1c; Table 1)^38^. Chimera cTEM-19m includes both segments of PSE-4 present in  either of the other chimeras, resulting in 19 substitutions on two active-site walls (S70 and Ω-loop walls) relative to TEM-1.

**Table 1:** Turnover rate constants for the representative hydrolysis of cephalothin by TEM-1, PSE-4 and their variants (See Table S9 for all kinetic parameters).

β-lactamase variant	Origin	*k*_cat_ (s^−1^)	*k*_cat_ fold variation relative to TEM-1
TEM-1^a^	native enzyme	84 ± 12	1.0
cTEM-17m^a^	engineered chimera	120 ± 8.0	1.4 (↑)
TEM-1(M69T)	deconvoluted variant	7.0 ± 4.0	12 (↓)
cTEM-18m(M69T)	deconvoluted variant	1.4 ± 0.3	60 (↓)
cTEM-2m	engineered chimera	2.8 ± 0.8	30 (↓)
cTEM-19m	engineered chimera	6.0 ± 0.5	14 (↓)
PSE-4^a^	native enzyme	0.80 ± 0.01	105 (↓)

^a^Data from^29^.

Natural evolution has explored at least 13 among the 19 mutated positions in the chimeras (Table S1)^39^. Among 32 natural variants of TEM-1 characterized to date for substitutions at position 68 and 69, Met68 can be mutated to Ile and Met69 to Ile/Leu/Val; the substitutions Met68Leu and the Met69Thr in the engineered cTEM-2m and cTEM-19m do not have natural counterparts^39^. Among the 17 substitutions resulting from the exchanged segment 150–190, only His153Arg and Met155Ile have been reported in six natural TEM-1 variants. The chimeras thus include active-site substitutions not yet observed in natural TEM-1 variants, yet those residues evolved in the homologous PSE-4 active site. Our previous reports of circular dichroism and thermal stability have shown that the three chimeras maintained the well-folded and stable nature of the parental enzymes^29,40^.

### Crystal structures are highly conserved

**Figure 2:**
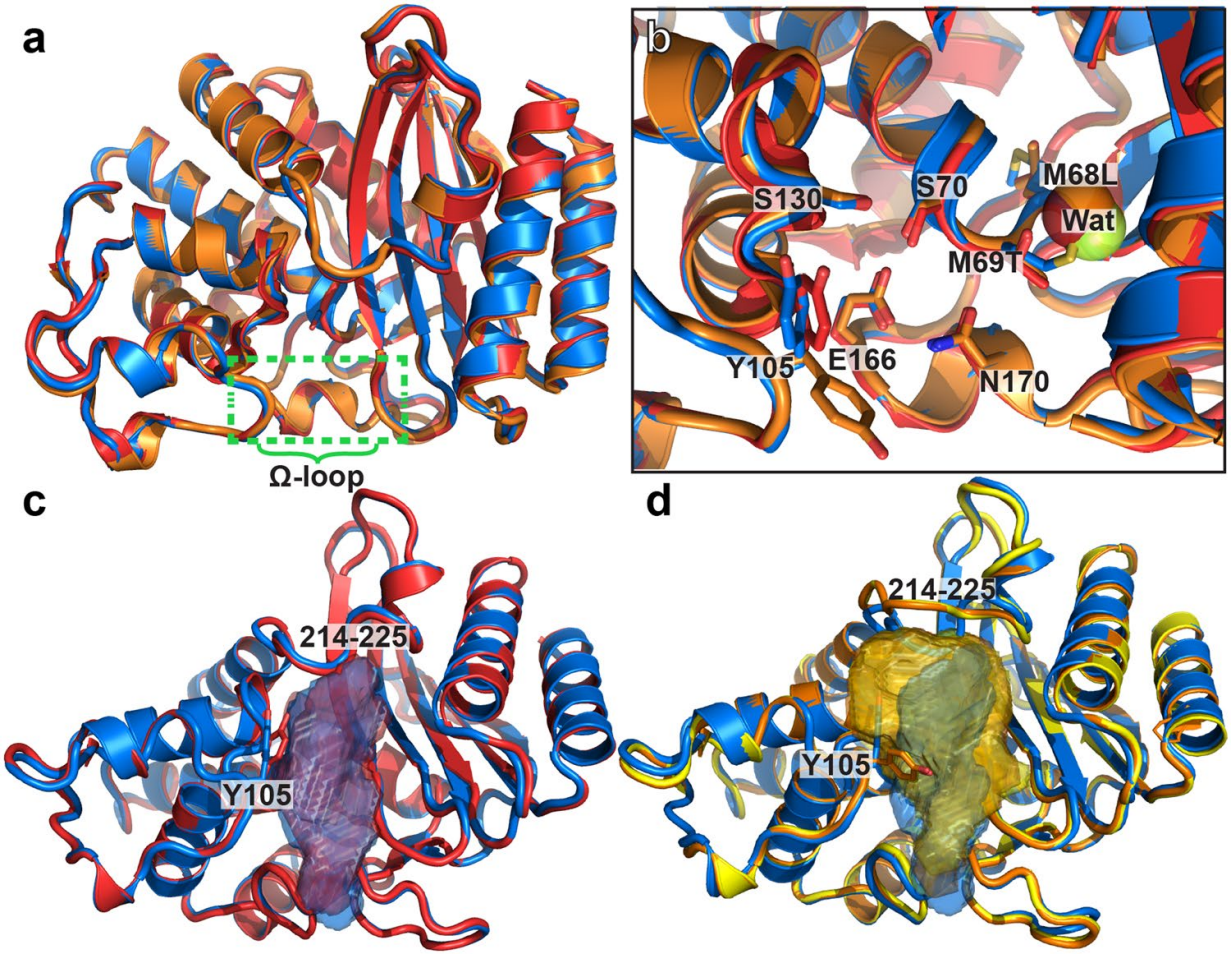
Crystal structures of cTEM-2m and cTEM-19m. (**a**) Backbone overlay of chimeras cTEM-2m (red; PDB ID: 4MEZ) and cTEM-19m (orange; PDB ID: 4R4S) over TEM-1 (blue; PDB ID: 1XPB). (**b**) Active-site view of (**a**). Wat: new water molecule in cTEM-2m (Wat519) and cTEM-19m (Wat478) relative to PSE-4 (green; Wat483). Tyr105 of cTEM-19m was modeled to the **m**-30° conformation, as opposed to the **t**80° conformation observed in TEM-1 and cTEM-2m. (**c**) Overlay of the active-site volume of TEM-1 and cTEM-2m. Orientation as in Fig. 1c. (**d**) Overlay of the active-site volume of TEM-1, cTEM-17m (yellow; PDB ID: 4ID4) and cTEM-19m. For residues with alternate conformations in the crystal structure, the conformer with the highest occupancy was illustrated. See Table S2 for data collection and refinement statistics.

Chimera cTEM-2m (PDB ID: 4MEZ, resolution 2.05 Å) and chimera cTEM-19m (PDB ID: 4R4S, 1.1 Å) maintained a native-like fold with no major modifications relative to the previously reported structures of TEM-1, PSE-4 and cTEM-17m (Fig. 2a; see Table S2 for the data collection and refinement statistics)^17,30,41^. We note that a water molecule near Met69Thr in cTEM-2m and cTEM-19m, and near Thr69 in PSE-4, fills the space otherwise occupied by Met69 in TEM-1 and cTEM-17m (Fig. 2b) and becomes an integral part of the active-site architecture.

Chimera cTEM-19m was most similar to cTEM-17m (Fig. 2b); both include segment 150–190 of PSE-4. Their active-site cavity volume (cTEM-19m: 741 Å^3^; cTEM-17m: 746 Å^3^; see Supplementary Methods) is nearly twice larger than the cavity of native TEM-1 (384 Å^3^), PSE-4 (427 Å^3^) and cTEM-2m (393 Å^3^). This results mainly from a rotation of Tyr105, in the Y105 gate-keeper wall. In cTEM-17m and cTEM-19m it is resolved in the **m**-30° conformation that swings the Tyr-phenoxy ring out of the active-site void, packing it against the cavity wall^42^. This opens and increases the active-site volume relative to the **t**80° conformation observed in all other native and mutant apoenzyme structures of TEM-1 and PSE-4 (Fig. 2b–d). We previously proposed that Tyr105 interconverts between the **m**-30° and **t**80°conformations^17,43^, consistent with this new crystal structure reflecting a dynamic event. The conformation trapped in the structures of cTEM-17m and cTEM-19m, but not in TEM-1, PSE-4 or cTEM-2m, may reflect an altered barrier to this conformational change.

The active-site volume is further increased in cTEM-17m and cTEM-19m by adoption of an open conformation of the active-site wall 214–218. It belongs to the 214–225 connector linking the all-α domain to the α/β domain (Fig. 2d). Interestingly, the structural changes that increase the cavity volume occur on active-site walls distal from the recombined 150–190 segment.

### Kinetic characterization of cTEM-2m and cTEM-19m

As a measure of function (*i.e*. hydrolysis of β-lactams), we report the macroscopic constants *K*_M_ and *k*_*cat*_ for hydrolysis of two penicillins and three cephalosporins by cTEM-2m and cTEM-19m and compare them to those of TEM-1, PSE-4 and cTEM-17m that we previously reported^29^. TEM-1 and PSE-4 hydrolyze benzylpenicillin and carbenicillin (penicillins), cephalothin and cefazolin (1^st^-generation cephalosporins) and cefotaxime (3^rd^-generation cephalosporin) with significantly different efficiencies. PSE-4 displays a 5-fold greater *k*_*cat*_ for carbenicillin turnover than TEM-1, justifying its classification as a carbenicillinase. It hydrolyzes penicillins (*k*_*cat*_/*K*_M_ 10^7^ M^−1^s^−1^) more efficiently than cephalosporins (*k*_*cat*_/*K*_M_ 10^2^–10^5^ M^−1^s^−1^); the difference is more marked than for TEM-1, mainly due to a greater drop (25- to 105-fold) in *k*_*cat*_ for hydrolysis of the cephalosporins (Table 1; see Table S9 for all kinetic parameters). The rate-limiting step for penicillins and cephalosporins differs: deacylation of the acyl-enzyme intermediate is rate-limiting for penicillin hydrolysis^44^, while for cephalosporins acylation is thought to be rate-limiting^45^ (Fig. 1d). By characterizing hydrolysis of both types of β-lactam antibiotics, we gain insight into any rate-limiting alteration of acylation or deacylation.

For all substrates investigated, the catalytic efficiencies of the three engineered chimeras were within one order of magnitude of TEM-1 or PSE-4 and were generally closer to TEM-1, consistent with these chimeras being more closely related to this native enzyme (Table 1; Table S9). This immediately illustrates that the catalytic machinery was largely unaltered by the recombination of TEM-1 and PSE-4. All chimeras displayed a catalytic efficiency *k*_*cat*_/*K*_M_ toward penicillin similar to TEM-1; none was as efficient as PSE-4 for carbenicillin (Table S9). This is consistent with having been selected for bacterial survival in the presence of ampicillin (a penicillin)^25,32^.

Greater variation of kinetic parameters was observed for hydrolysis of cephalosporins. The inclusion of 17 substitutions left cTEM-17m essentially unaltered relative to TEM-1, but inclusion of the substitutions Met68Leu/Met69Thr in cTEM-2m and cTEM-19m dropped *k*_*cat*_ 1 to 3 orders of magnitude, to levels similar to PSE-4. This is illustrated by the results of cephalothin hydrolysis (Table 1). Furthermore, the *K*_M_ values for cephalosporins both increased and decreased in the chimeras without a clear trend, expanding the range of *K*_M_ values to a 10-fold difference (Table S9). This variation in productive binding may result from the more voluminous substituents of cephalosporins than penicillin: their binding may be more highly modulated by the sequence changes and dynamic effects that may follow^45^.

We deconvoluted the two substitutions that had the greatest effects on cephalosporin hydrolysis (Fig. 1). Inclusion of the Met69Thr substitution alone in TEM-1 (TEM-1(M69T)) or in cTEM-17m (cTEM-18m(M69T)) reduced both *k*_*cat*_ (up to 80-fold) and *K*_M_ (up to 40-fold; Table 1 and Table S9). As a result, catalytic efficiency (*k*_*cat*_/*K*_M_) for cephalosporin hydrolysis remained similar to TEM-1. The Met68Leu substitution had little effect on the kinetics (<6-fold variation with the exception of *k*_*cat*_^CTX^, 12-fold decrease). Our results demonstrate that inserting Thr69 from PSE-4 into TEM-1, immediately beside the conserved catalytic Ser70, is sufficient to switch cephalosporin hydrolysis from TEM-1-like to less efficient PSE-4-like. Overall, both for penicillin and cephalosporin hydrolysis, the engineered chimeras display kinetics similar to the native proteins they originate from, indicating that the rate-limiting steps have not been significantly altered.

### Validating MD simulation methodology to access ns-µs timescale motions

Having determined that the structure and function of these five related proteins are subtly altered by active-site recombination, we determined the continuum of fast-to-slow dynamics (ps to ms) because they describe events leading to, and including, the rate-limiting steps during catalysis. Although β-lactamases are moderate-sized proteins (∼30 kDa), their size is sufficient to render the NMR observation of specific timescales – particularly the ns-μs timescale – difficult. Furthermore, NMR backbone relaxation experiments leave an information gap relative to side-chain motions because these are loosely inferred from the backbone motions. Those experiments also leave gaps where prolines reside and where assignment is incomplete due to overlapping N-H resonances or if resonance broadening drives signals into the background.

In contrast, molecular dynamics (MD) simulations provide a full atomistic model of ps-µs timescale motions for proteins of moderate size. Our first goal was to validate whether MD simulations could reliably predict the *S*^2^ order parameters previously determined by NMR for TEM-1, PSE-4 and cTEM-17m (Tables S3 to S5)^27,29^. *S*^2^ characterizes motions on the ps-ns timescale, indicating whether the peptide N-H bonds are rigid (*S*^2^ near 1) or flexible (*S*^2^ < 0.5). For each protein, three independent 2 µs MD simulations were performed.

For all proteins, the mean *S*^2^ calculated from MD simulations compared favorably to the NMR data (Δ*S*^2^_MD-NMR_ within 0.03) (Table S3). The per-residue *S*^2^ value was between the average ±1 S.D. (*i.e*. 0.81 to 0.94) for 87% of residues, and 73% of all residues were in excellent agreement (per residue Δ*S*^2^_MD-NMR_ ≤ 0.05). Greater *S*^2^ divergence was observed for some surface-exposed residues, yet the agreement was good even for the most dynamic residues (*S*^2^ < 0.81) where one might expect greater divergence (Fig. S1**)**. Despite isolated variations, the excellent agreement obtained for TEM-1, PSE-4 and cTEM-17m β-lactamases allowed us to conclude that we can confidently extract *S*^2^ order parameters from the MD simulations of cTEM-2m and cTEM-19m β-lactamases.

For the reasons described above, and particularly as a result of significant resonance broadening, NMR *S*^2^ data are missing for crucial active-site residues from the following active-site walls in one or more proteins: S70, 129–132 SDN, Ω-loop, 214–218 and 234–244 (Table S5)^40,46–48^. Consequently, the complete *S*^2^ datasets extracted from the MD simulations of TEM-1, PSE-4 and cTEM-17m served for all further analyses.

### Fast motions: dynamics of the β-lactamase system on the ps-ns timescale

**Figure 3:**
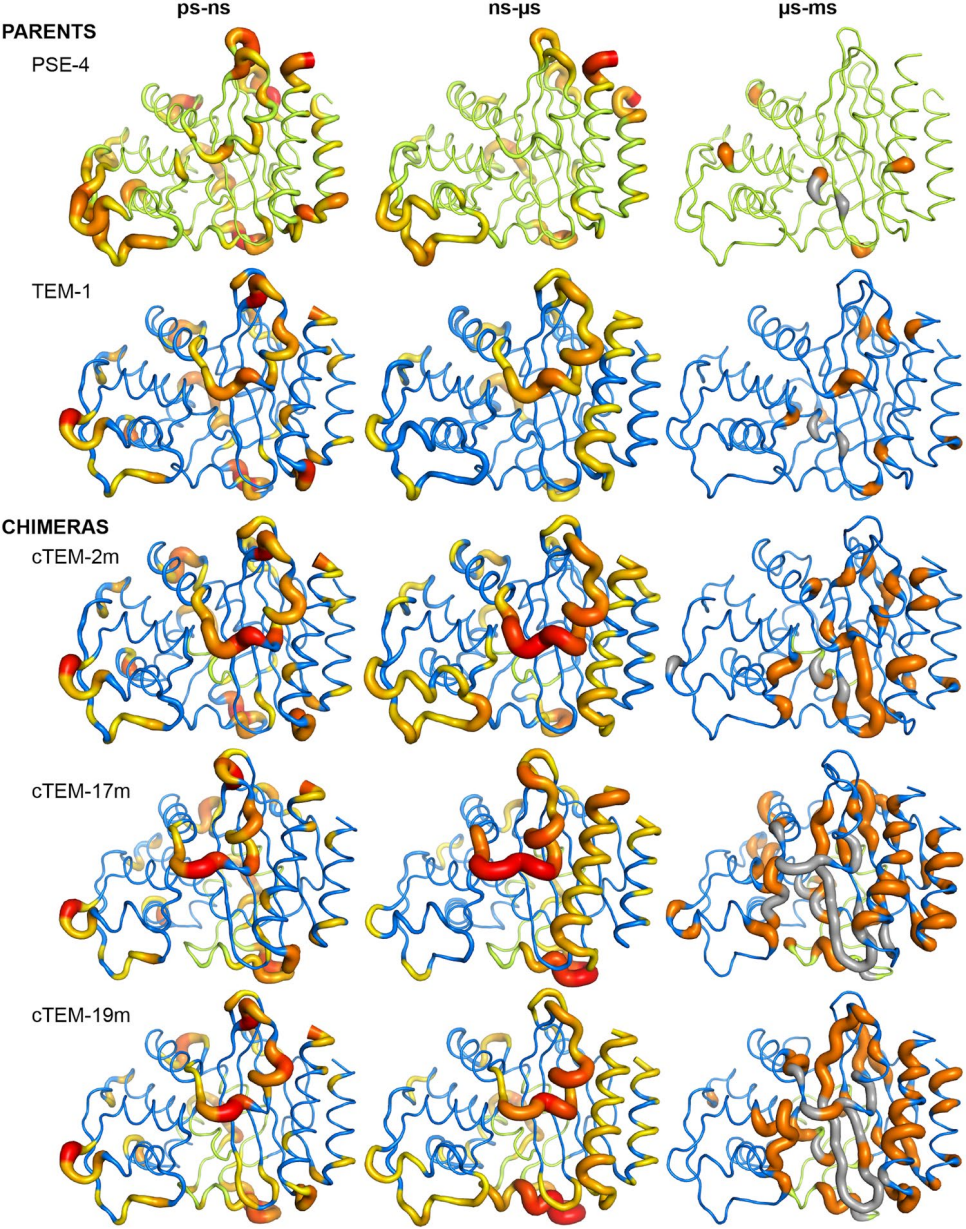
β-Lactamase dynamics on the ps to ms timescales. Dynamics of the parents TEM-1 (blue, PDB ID: 1XPB), PSE-4 (green, PDB ID: 1G68) and the laboratory-evolved chimeras cTEM-2m (PDB ID: 4MEZ), cTEM-17m (PDB ID: 4ID4) and cTEM-19m (PDB ID: 4R4S) colored blue/green according to the parental origin of each segment, as in Fig. 1. Left: Dynamic residues on the ps-ns timescale are given by the MD-derived *S*^2^ order parameter for the amide NH. Residues with *S*^2^ lower than protein average *S*^2^ are colored and thickened on a scale of yellow (<0.85; less dynamic) to red (≤0.7; more dynamic). Middle: Dynamics on the ns-µs timescale are given by the Cα-RMSF calculated from triplicate 2 µs MD simulations. Residues with Cα-RMSF above the protein average Cα-RMSF are colored and thickened on a scale of yellow (>0.10 nm; less dynamic) to bright red (≥0.25 nm; more dynamic). Right: Dynamics on the µs-ms timescale were monitored by CPMG-NMR on the ^1^H-^15^N vector. Residues showing dispersion curves with Δ R_2_ ≥ 7.0 s^-1^ at 800 MHz are colored orange. NMR unassigned residues are colored gray. Raw data are presented in Tables S2 to S7 and Figs S1 to S4.

All β-lactamases showed high *S*^2^ inferring high rigidity on the ps-ns timescale. Comparison of *S*^2^ values showed that the core of each β-lactamase under study was rigid. The more flexible residues were in surface loops, including active-site loops (Fig. 3). The hinge leading into the Ω-loop showed fast dynamics in all proteins (average *S*^2^_155–160_ = 0.79 to 0.81), as did the tip of the Ω-loop (*S*^2^_171–178_ = 0.78 to 0.80) (Table S8). The active site wall 214–218 was more dynamic in TEM-1 (*S*^2^_214–218_ = 0.80) and in the three chimeras (*S*^2^_214–218_ = 0.76 to 0.78) than in the more distantly related PSE-4 (*S*^2^_214–218_ = 0.85). High standard deviations were observed in certain regions, particularly for the three chimeras (Fig. S1). The standard deviation derived from simulations reveals that a residue explored states with high and low *S*^2^ over the course of the simulated trajectories, similarly to NMR where standard deviation on the model-free fitting of *S*^2^ reflects the extent of sub-state sampling. As the *S*^2^ calculation was performed on multiple 10 ns segments over the total 6 µs simulated trajectories, the standard deviation reflects conformational changes that may occur on timescales longer than 10 ns. Low simulated *S*^2^ and high standard deviations allowed identifying regions that may exhibit increased motions on slower timescales. For example, the active-site wall 234–244 was rigid in all proteins (*S*^2^ = 0.86–0.88), yet in cTEM-17m and cTEM-19m a higher per-residue standard deviation suggests motions occurring on a slower timescale. Those slower motions were confirmed and characterized (Figs S1 and S2). *S*^2^ in those regions generally could not be characterized by NMR due to overlapping/broad peaks, further demonstrating the value of the simulations.

### Dynamics on the intermediate (ns-µs) timescale

To investigate the intermediate timescale of motions, we extracted the Cα root mean square fluctuation (RMSF) from the molecular dynamics simulations (Fig. S2 and Table S6). As observed for fast motions, the core of each β-lactamase was rigid, while the surface loops were more flexible (Fig. 3). TEM-1 and the chimeras showed significantly more ns-µs dynamic residues than PSE-4, in agreement with the higher sequence similarity of the chimeras to TEM-1. The substitution of up to 19 amino acids in TEM-1 did not markedly alter ns-µs dynamics.

The pattern of active-site wall dynamics was essentially the same at both timescales (Table S8). The leading hinge and tip of the Ω-loop showed above-average dynamics in all proteins (RMSF = 0.12–0.23 nm, relative to protein averages of 0.07–0.10 nm), and all but PSE-4 were dynamic in the 214–218 wall (RMSF = 0.15–0.25 nm; 0.07 nm for PSE-4). In addition, chimeras cTEM-17m and cTEM-19m showed above-average RMSF for the entire Ω-loop (RMSF = 0.14 and 0.17 nm, respectively; 0.07–0.10 nm for the others). Finally, the Y105 active-site wall of cTEM-2m had an RMSF of 0.14 ± 0.05 nm (0.8–0.11 nm for the others), indicating ns-µs motions unique to this chimera.

As observed for *S*^2^ on the ps-ns timescale, above-average per-residue standard deviation of several active-site walls was observed, and was overwhelmingly linked to increased RMSF (Fig. S2). Again, increased standard deviation in the observed RMSF reflected conformational changes slower than the 6 μs total simulation time, as described below.

### Slow motions: dynamics on the µs-ms timescale probed by ^15^N CPMG NMR

The timescale describing the slowest motions (µs-ms) was probed by NMR protein backbone methods. This timescale remains somewhat beyond the reach of current computational simulation capacity for a system constituted of several proteins of moderate size. Comparison of backbone resonance assignments^40,46^ revealed minimal chemical shift (σ) differences except where there are sequence differences, as expected (Fig. S3). Nonetheless, assignments were significantly less complete for cTEM-17m and cTEM-19m (91–92%) than TEM-1, PSE-4 and cTEM-2m (98–99%). This results from NMR resonance broadening, preventing assignment of residues that exhibit motions on a timescale slower than that probed^49–51^.

Among the 26 dynamic residues in cTEM-2m, we find 7 of the 11 dynamic residues of TEM-1. They are located in the active site and in the α/β domain, the latter containing the majority of the new dynamic residues of cTEM-2m. Thus, the slow motions of TEM-1 were generally maintained while additional residues acquired ms conformational exchange. Considering that the substitutions in cTEM-2m (residues 68–69) are located within the active site, it is surprising that the new dynamics should extend specifically into the α/β domain but not the all-α domain, and range widely to residues and regions that are not associated by distinct H-bonding or other networks (Fig. 3).

Interestingly, there was no difficulty in assigning the cTEM-2m residues 236–246 that were unassigned in cTEM-17m and cTEM-19m, yet that region of cTEM-2m is highly dynamic. This suggests that the timescale of dynamics differs between the chimeras. Dynamic residues in cTEM-2m border and extend throughout the active-site wall 234–244 (Ile231, Lys234, Gly238 and residues 241–246), while TEM-1 showed a single dynamic residue there (Arg241). The dynamic region is further extended in cTEM-17m and cTEM-19m, linking up the dynamic 234–244 and 214–218 active-site walls; only three non-dynamic residues (and a Pro, which cannot be probed by ^15^N CPMG NMR) interrupt the 214–244 dynamic stretch.

This extensive region is distal to both sites of recombination, yet the recombination of region 66–73 (cTEM-2m), and of region 150–190 (cTEM-17m), each produce increased dynamics therein. In particular, recombination of region 150–190 dramatically heightened the extent of the dynamics, as observed by extensive NMR peak broadening (unassigned residues) in addition to 60 assigned dynamic residues. Among these, 35 were identical in cTEM-17m and cTEM-19m, thus are attributable to the recombination of region 150–190. Surprisingly, 12 of the dynamic residues in cTEM-17m and 18 in cTEM-19m were also dynamic in cTEM-2m. The two distinct recombination events thus triggered partly overlapping increases in slow dynamics in a region distal to each of the recombined regions. However, recombination of region 150–190 also resulted in new slow dynamics in the all-α domain, resulting in a distinctly different pattern of new slow dynamics than the recombination of region 66–73.

### Functional β-lactamases tolerate different frequencies of slow motions within the active site

**Figure 4:**
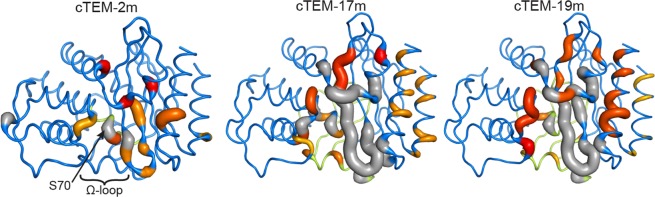
Global exchange rates (*k*_ex_) of cTEM-2m, cTEM-17m and cTEM-19m. The *k*_ex_ of the fitted regions are colored and thickened on a scale of yellow (300 s^−1^) to red (1500 s^−1^) on the crystal structure coordinates of cTEM-2m (PDB ID: 4MEZ), cTEM-17m (PDB ID: 4ID4) and cTEM-19m (PDB ID: 4R4S). NMR unassigned residues are colored dark gray. The fitted regions were the *N*-terminal helix (H1, residues 28–41), S70 wall (residues 69–72), Y105 wall (residues 105–106), SDN wall (residues 128–132), Ω-loop wall (residues 166–170), the domain connector (214–225), 234–244 wall and the *C*-terminal helix (H11, 272–288).

The frequency of the slow dynamics, or transition rate between distinct conformations, was determined according to the exchange rate constant (*k*_ex_) to inform on the sampling rate of interconverting conformations. The per-residue *k*_ex_ calculated for ^15^N-CPMG relaxation curves observed at 800 MHz and 500/600 MHz (Table S7) generally overlapped for stretches of residues, allowing calculation of the global fit for *k*_ex_ per wall (Table S8). Interestingly, the exchange rates *k*_ex_ for the numerous assigned, dynamic residues in the 214–244 region of cTEM-17m and cTEM-19m are comparable to their homologs TEM-1 and cTEM-2m (Fig. 4, Table 2). However, the frequency of motions is not fully conserved since the unassigned residues in cTEM-17m and cTEM-19m are indicative of an exchange rate slower than the millisecond. The α/β domain therefore tolerates important modifications in the number and exchange rates of the dynamic residues, while maintaining catalytic proficiency.

**Table 2:** Global exchange rates (*k*_ex_) calculated for the active-site walls.

Active-site wall	*k*_ex_ (s^−1^)				
	TEM-1	PSE-4	cTEM-2m	cTEM-17m	cTEM-19m
S70	—	—	940 ± 250	480 ± 110	670 ± 140
Y105	—	—	ND	300 ± 90	1,220 ± 230
SDN	ND	—	480 ± 210	780 ± 270	980 ± 200
Ω-loop	—	2,020 ± 810	—	590 ± 130	520 ± 110
214–225	1,390 ± 370	—	1,340 ± 780	1,100 ± 440*	1,130 ± 420*
234–244	650 ± 30	—	610 ± 220	910 ± 210*	800 ± 250*

* indicates regions that include multiple unassigned residues.

ND: *k*_ex_ was not determined as a result of large error on the calculated fit.

The all-α domain active-site walls (S70, Y105, SDN and Ω-loop walls) of cTEM-17m and cTEM-19m showed extensive ms dynamics. A new stretch of dynamic residues was observed in their Ω-loop wall, with similar *k*_ex_ (Table S8). Furthermore, new dynamic residues in the S70 and S130 active-site walls of the three chimeras differed by less than 2-fold in *k*_ex_. Thus, recombination of region 150–190 in cTEM-17m and cTEM-19m and, to a lesser extent, region 66–73 in cTEM-2m, increased the number of dynamic residues on the all-α domain side of the active site, with similar *k*_ex_ regardless of the recombination.

The Y105 wall displayed slow dynamics in the three chimeras, with a 4-fold difference in *k*_ex_ for cTEM-17m (300 ± 90 s^−1^) and cTEM-19m (1,220 ± 230 s^−1^). Y105 participates in substrate stabilization^52,53^; a variation in slow dynamics of this residue could be expected to have a functional implication. However, this pattern does not correlate with the rate constants for cephalosporin hydrolysis (Table 1; Table S9), despite those *k*_ex_ being on the timescale of cephalosporin turnover (*i.e*. TEM-1 *k*_*cat*_^CF^ = 84 s^−1^). Altered dynamics of the Y105 wall thus do not have an observable impact on function. In general, the new dynamic exchange rates observed by CPMG NMR in all regions of cTEM-2m, cTEM-17m and cTEM-19m were between 300 s^−1^ and 2,000 s^−1^, a rate comparable to turnover (Table 1; Table S9). These results do not preclude potential alterations of the slower-than-observed dynamics at residues that were not NMR-assignable. The unassigned Ser70, in particular, may be relevant to the observed alterations in kinetics of cephalosporin hydrolysis.

## Discussion

### The continuum of fast-to-slow dynamics in relation to function in a β-lactamase system

Despite differences observed in slow dynamics, such as observations made for residue Tyr105 (altered crystal conformation, new relaxation dispersion, varied *k*_ex_), the engineered chimeras are highly functional, their kinetic parameters ranging near those of the native TEM-1 and PSE-4. This suggests that the rate-limiting steps for hydrolysis of penicillins and of cephalosporins are not dependent on the window of motions we have examined, including the slow motions that are near the timescale of catalytic turnover.

On the timescale of the fastest motions examined, ps-ns motions in TEM-1 and PSE-4 and the chimeras were almost identical and exhibited a rigid core with modest surface loop dynamics (Fig. 3)^17,29,53^. On the intermediate ns-µs timescale, the enzymes revealed a “dynamic personality”: while the surface loops were mildly flexible in TEM-1, they were less flexible in the three chimeras, and almost rigid in PSE-4. Thus, the dynamics of the chimeras are mainly TEM-1-like, yet are skewed toward the properties of PSE-4, reflecting their relative sequence similarity. On the timescale of the slowest motions examined (µs to ms), TEM-1 and PSE-4 exhibited few dynamic residues^17^. In sharp contrast, the chimeras displayed widespread increases in slow dynamics within the protein core, the pattern of which depended on the recombined region. In addition, faster motions with high standard deviations were accompanied by observation of slower motions in the same regions; as a result, we observe a continuum of motions.

The impact of acquiring timescale-specific dynamic data has been shown in studies of ubiquitin: acquiring ns-µs timescale dynamics was essential to correlate flexibility and formation of complexes with different ligands^54^. Here, the importance of observing multiple dynamic timescales is vividly illustrated for β-lactamase enzymes by observing the active-site wall 234–244. It displayed no fast motions in any of the β-lactamases under study; studies limited to the ps-ns-µs regime would thus conclude to a rigid wall. Nonetheless, RMSF values (ns-µs) and their standard deviation hinted at new slow dynamics. Indeed, this entire wall was unassigned by NMR, and slow dynamics were observed in flanking residues as a result of both patterns of sequence substitution. Similarly, the gatekeeping Tyr105 showed no fast dynamics yet exhibited new, slow dynamics in the three chimeras as a result of either of the substitutions. Since the spatial relation is different between each active-site wall and the TEM-1/PSE-4 recombinations 66–73 or 150–190 (Fig. 1c), we demonstrate that different recombination sites within the active-site cavity have triggered a variety of slow dynamic patterns throughout all faces of the active site, and those motions are all compatible with native-like catalytic activity.

### Do crystallographic B-factors reflect the observed protein dynamics?

**Figure 5:**
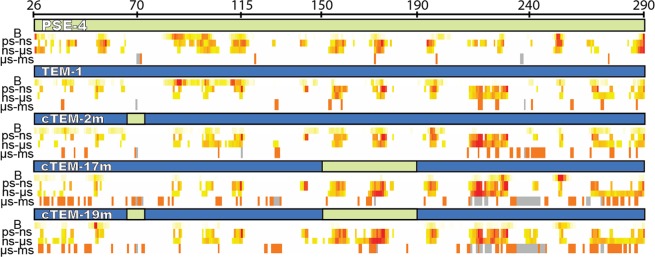
Distribution of motions in the β-lactamases over the 3 observed timescales in relation to the linear sequence and crystallographic B-factors. The sequence blocks originating from TEM-1 (blue) and PSE-4 (green) are colored in cTEM-2m, cTEM-17m and cTEM-19m according to their parental origin as in Fig. 1. Crystallographic B-factors (B) above the protein average are colored on a scale of yellow (average + 1 st. dev.) to bright red (highest B-factor for that protein) (PDBs 1G68, 1XPB, 4MEZ, 4ID4 and 4R4S, respectively). The ps to ms motions are shown in yellow (less dynamic) to red (more dynamic), as in Fig. 3. NMR unassigned residues are colored gray.

Although crystallographic B-factors do not directly inform on the timescale of motions and may reflect phenomena that are unrelated to solution protein dynamics^35,36^, there are instances where high B-factors are interpreted as definitive protein motions^22,33,34,55^. We verified whether high B-factors could offer insight into dynamics of the system under investigation (Fig. 5).

A few high B-factors (above the protein average) are reported in the crystal structures of TEM-1, PSE-4 and cTEM-2m (1.9–2.05 Å resolution). When present, they generally correlated with some of the ps-ns and ns-µs motions we identified, but showed no significant correlation with the slow motions (Fig. 5). Although the few above-average B-factors in cTEM-17m and cTEM-19m (1.05 and 1.1 Å resolution) are predominantly located in the highly dynamic 214–244 region, it is striking that the high prevalence of slow motions that characterize those two chimeras is in no way reflected by a greater number of higher B-factor values. We thus see no convincing evidence that high B-factors in these crystal structures reflect protein motions on the slow timescale, or yet even slower motions in the regions that could not be assigned in our solution experiments.

Clearly, protein dynamics in crystals and solution cannot be identical. Although direct comparison of crystallographic B-factors with solution NMR order parameters can be limited due to the distinctly different nature of these observables^56^, developments in solid-state NMR^57,58^ and comparative MD simulations^59,60^ have provided insights regarding protein dynamics in solution *vs* crystals: in general agreement with our observations of ps-ns and ns-µs motions, the crystal lattice has little impact on sub-microsecond dynamics. Recent studies^61–63^ indicate that crystal packing effects result in pronounced consequences on slow dynamics. Exchange rates are slower relative to solution, and the rates as well as the relative populations of the exchanging states differ importantly between crystal forms^61^.

In the β-lactamases under study, 20–24% of the protein residues are directly involved in crystal contacts which may inhibit larger motions (Table S10). As a result, the B-factors of these residues and, presumably, of their spatially neighboring residues, may not accurately reflect their slow motions in solution. Complicating matters, crystal contacts do not necessarily prevent mobility. This is clearly evident in our high-resolution structures in which the 214–244 stretch was observed in alternate conformations at a crystal contact region. To parse out innate dynamism, one also needs to rule out potential static disorder caused by crystal packing which could artifactually inflate B-factors. In addition to crystal packing effects, noise from lattice disorder and dependence on the method of structure refinement are some of the limitations of B-factors when applied to study protein flexibility and motion^55^. In light of these considerations, the results of the current study caution against overinterpretation of crystallographic B-factors: whether slow dynamics on longer (µs-ms) timescales are sustained in crystals is only now beginning to be investigated.

### Protein motions are both conserved and divergent in engineered β-lactamases

Two key observations came to light upon aligning the motions observed in this protein system over the three timescales that were monitored (Fig. 5). First, the greatest dynamic changes occurred outside of the substituted regions, hinting at a dynamic network in β-lactamases. Second, the dynamics on each timescale varied significantly for any given residue. We thus directly observe that one cannot infer dynamics outside of timescales that have been probed^64^. In addition, crystallographic B-factors were only weakly predictive of in-solution motions, particularly those on the slow timescale.

The engineered chimeras are the result of functional selection from a vast pool of recombined variants^25,32^. The combination of conserved rigidity on the fast timescale and new, slow motions argues that alteration of the fast motions is not functionally tolerated. Although they likely evolved separately, our results are strikingly similar to point mutants of *Bacillus cereus* metallo-β-lactamase II: fast dynamics were conserved and important variations in slow active-site dynamics were observed^65^. β-Lactamases are specialized, highly evolvable antibiotic resistance enzymes that require great adaptability to the consequences of sequence variation to ensure bacterial survival. We speculate that the observed tolerance to diverse slow active-site motions may facilitate evolution and promote functional diversity, particularly substrate promiscuity^11,23,24^. A similar hypothesis has been made of a resurrected Precambian β-lactamase where conformational flexibility was beneficial to develop new function^66^.

In conclusion, we have demonstrated that the links between sequence variation, catalytic function and motions on different timescales can differ vastly in β-lactamases. A limited inspection of protein dynamics could lead to mistaken conclusions on the impact of dynamic conservation or variation, and the field will welcome continuing developments in wet-lab and computational methods for determination of protein dynamics. Although these are early days in our understanding of the functional implications of protein dynamics, we can aspire to engineer function through motions.

## Materials and Methods

Protein expression and purification was performed as previously reported^17,29,40^. Crystals were grown at 22 °C in hanging drops with a 1:1 ratio of the protein and reservoir solutions. Diffraction data were collected at the Canadian Macromolecular Crystallography Facility Beamline 08ID-1 or with a Rigaku MicroMax 007 HF X-ray generator and a Rigaku Saturn 944 HG CCD detector. Initial phases were calculated by molecular replacement, with PDB ID: 1ZG4. Iterative rounds of manual model building were performed. The active-site volume was estimated using 3 V: Voss Volume Voxelator and probes of 1.5 Å and 8 Å radius. For energy minimization, MD simulations and analysis, GROMACS 5.0.1 was used with the AMBER99SB-ILDN force field^67,68^. Three 2 µs simulations with 1 ps compressed and 100 ps full precision trajectories for each of the five proteins was acquired. *S*^2^ order parameters were calculated applying the model-free approach^69^. The Cα-RMSF was calculated for each residue and converted into B-factors. NMR experiments were performed as previously reported^17,40^. [^15^N] and [^2^H,^15^N]-labeled samples were characterized using ^15^N TROSY relaxation-compensated Carr-Purcell-Meiboom-Gill (rcCPMG) experiments^70^ on Agilent 800 MHz, 600 MHz and 500 MHz NMR spectrometers calibrated to 31.5 °C. Residue fits and model analyses were performed using the full single-quantum ^15^N-CPMG equation^71^. Substrate hydrolysis was monitored according to initial steady-state velocities. Detailed experimental procedures can be found in Supplementary Methods.

## Supplementary information

Supplementary Information file

Dataset 1

Dataset 2

Dataset 3

Dataset 4

## Data Availability

The atomic coordinates have been deposited in the Protein Data Bank, www.pdb.org, under the accession codes 4MEZ (cTEM-2m) and 4R4S (cTEM-19m).
