# Supplementary material for: The Structural Dynamics of Engineered β-Lactamases Vary Broadly on Three Timescales yet Sustain Native Function
Source: Sci Rep. 2019 Apr 30;9:6656. doi: 10.1038/s41598-019-42866-8 (PMC6491436; doi:10.1038/s41598-019-42866-8)
Supplement: Supplementary file 1 — Supplementary Information file [file 41598_2019_42866_MOESM1_ESM.pdf]

TITLE

The Structural Dynamics of Engineered β-Lactamases Vary Broadly on Three Timescales yet Sustain Native Function.

RUNNING TITLE

Structural Dynamics of Engineered β-Lactamases

AUTHORS

S. M.-C. Gobeil, M. C. C. J. C. Ebert, J. Park, D. Gagné, N. Doucet, A. M. Berghuis, J. Pleiss and J. N. Pelletier.

Supplementary Information

1. SUPPLEMENTARY TABLES AND FIGURES

Supplementary Table S1. Natural TEM variants at the 19 positions that differ between TEM-1 and cTEM-19m.

Supplementary Table S2. Data collection, refinement statistics, Cα-RMSD and secondary structure matching RMSD comparison for crystal structures of the chimeras cTEM-2m (PDB 4MEZ) and cTEM-19m (PDB 4R4R and 4R4S).

Supplementary Table S3. Dynamics of the naturally evolved β-lactamases TEM-1 and PSE-4 and the chimeras cTEM-2m, cTEM-17m and cTEM-19m. Protein-averaged, squared generalized order parameters (S^2) obtained by MD simulations and NMR relaxation for the amide NH bonds and Cα-RMSF extracted from the MD simulations.

Supplementary Table S4. Cα-RMSD between the average structure from each 2 µs simulation and the crystal structure used for that simulation.

Supplementary Table S5. Per residue S^2 extracted from MD simulations for TEM-1, PSE-4, cTEM-2m, cTEM-17m and cTEM-19m compared to the NMR-determined S^2 for TEM-1, PSE-4 and cTEM-17m.

Supplementary Table S6. Per residue Cα-RMSF extracted from triplicate MD simulations of 2 µs each for TEM-1, PSE-4, cTEM-2m, cTEM-17m and cTEM-19m.

Supplementary Table S7. Differences in measured R_2 (1/ τcp) at fast (τcp = 0.625 ms) and slow (τcp = 10 ms) pulsing rates (ΔR_2 values ≥ 7s^{-1}), derived from TROSY-based ^{15}N-CPMG relaxation dispersion experiments, and unassigned residues (NA) for the chimeras cTEM-2m and cTEM-19m.

Supplementary Table S8. Comparison of the dynamism of the active-site walls using the S^2 (ps-ns), Cα-RMSF (ns-µs) and k_{ex} (µs-ms) on the different timescales.

Supplementary Table S9. Kinetic parameters for hydrolysis of the cephalosporins cephalothin (CF), cefazolin (CZ) and cefotaxime (CTX) **and penicillins** benzylpenicillin (BZ) and carbenicillin (CB) by the parental class A β-lactamases TEM-1 and PSE-4, the chimeras cTEM-2m, cTEM-17m and cTEM-19m and the deconvoluted mutants in the cTEM-17m context (cTEM-18m(M68L) and cTEM-18m(M69T)) and the TEM-1 context (TEM-1(M68L) and TEM-1(M69T)).

Supplementary Table S10. Crystal contacts for each of the β-lactamases under study.

Supplementary Figure S1. Sequence relation in the exchanged regions of the chimeras to the parental TEM-1 and PSE-4 β-lactamases and generalized squared order parameters (S^2) along the protein sequence for the amide NH bonds.

Supplementary Figure S2. Cα-RMSF derived from triplicate 2 µs MD simulations.

Supplementary Figure S3. Effect of the recombination of regions 66-73 and 150-190 on the backbone chemical shifts.

Supplementary Figure S4. ^{15}N-CPMG relaxation dispersion curves for (a) cTEM-2m and (b) cTEM-19m at 800 MHz and 500 or 600 MHz.

2. SUPPLEMENTARY MATERIALS AND METHODS

3. SUPPLEMENTARY REFERENCES

Supplementary Table S1. Natural TEM variants at the 19 positions that differ between TEM-1 and cTEM-19m [1].

TEM-1	cTEM-19m	TEM variant	
M68	L	I	TEM-192
M69	T	I	TEM-32, TEM-37, TEM-40, TEM-159, TEM-182
		L	TEM-33, TEM-35, TEM-39, TEM-45, TEM-50, TEM-77, TEM-80, TEM-81, TEM-83, TEM-109, TEM-125, TEM-154, TEM-158, TEM-169, TEM-185, TEM-189, TEM-190
		V	TEM-34, TEM-97, TEM-36, TEM-38, TEM-78, TEM-82, TEM-151, TEM-152, TEM-160
A150	D	-	
H153	R	R	TEM-21, TEM-56, TEM-112, TEM-215
N154	Q	-	
M155	I	I	TEM-156, TEM-199
H158	K	N	TEM-127
V159	E	-	
W165	I	C	TEM-83
		G	TEM-169, TEM-190
		L	TEM-182
		R	TEM-39, TEM-78, TEM-125, TEM-185
E168	D	K	TEM-193
A172	G	-	
I173	K	V	TEM-132
P174	L	-	
N175	G	H	TEM-193, TEM-194, TEM-195
		I	TEM-138
E177	T	-	
M182	L	T	TEM-20, TEM-32, TEM-43, TEM-52, TEM-63, TEM-72, TEM-87, TEM-88, TEM-91, TEM-92, TEM-93, TEM-94, TEM-106, TEM-107, TEM-113, TEM-124, TEM-126, TEM-131, TEM-135, TEM-149, TEM-153, TEM-159, TEM-161, TEM-165, TEM-177, TEM-184, TEM-205, TEM-211, TEM-220
A184	K	V	TEM-116, TEM-157, TEM-162, TEM-181, TEM-187, TEM-197, TEM-205
M186	I	V	TEM-193, TEM-194, TEM-195
T188	S	K	TEM-148
		N	TEM-193

Supplementary Table S2. (a) Data collection and refinement statistics and (b) Cα RMSD and secondary structure matching RMSD (in parentheses) comparison for crystal structures of the chimeras cTEM-2m (PDB 4MEZ) and cTEM-19m (PDB 4R4R and 4R4S). Although the classical β-lactamase inhibitor tazobactam was included during crystallization for cTEM-19m (PDB 4R4S), no corresponding electron density was observed. The crystal of cTEM-19m grown in the absence of tazobactam (PDB 4R4R) provided a nearly identical structure (Cα-RMSD 0.04 Å).

(a)

	**cTEM-19m**		**cTEM-2m**
	**PDB 4R4R**	**PDB 4R4S**	**PDB 4MEZ**
**Data collection**			
Space group	P 21 21 21	P 21 21 21	P1
Unit-cell parameters (Å)			
a	36.69	36.71	34.33
b	58.79	58.71	55.07
c	109.83	109.77	77.68
Resolution (Å)	1.20	1.10	2.05
Completeness (%)	98.3 (91.6)	98.3 (84.5)	99.2 (97.2)
Average redundancy	3.0 (2.6)	5.6 (3.2)	7.9 (5.9)
I/σ(I)	16.3 (4.3)	21.3 (3.4)	11.8 (3.5)
R-merge (%)	3.6 (23.6)	4.1 (28.6)	15.2 (39.2)
**Refinement**			
R-work (%)	10.5	10.4	19.2
R-free (%)	13.8	12.8	24.8
No. of water	408	464	346
r.m.s. deviations			
Bond lengths (Å)	0.021	0.021	0.020
Bond angles (°)	2.0	2.0	2.0

(b)

	TEM-1 *(1XPB - 1.9 Å)*	***cTEM-2m*** *(4ID4 - 1.05 Å)*	***cTEM-19m*** *(4R4S - 1.12 Å)*
TEM-1 *(1XPB - 1.9 Å)*	--	0.71 (0.42)	0.93 (0.51)
PSE-4 *(1G68 - 1.95 Å)*	1.35 (1.01)	1.42 (1.12)	1.44 (1.11)
cTEM-17m *(4MEZ - 2.05 Å)*	0.93 (0.55)	0.80 (0.59)	0.14 (0.28)

*Crystal structure resolution indicated next to PDB code.

Supplementary Table S3. Dynamics of the naturally evolved class A β-lactamases TEM-1 and PSE-4 and chimeric β-lactamases cTEM-2m, cTEM-17m and cTEM-19m. Protein-averaged, squared generalized order parameters (S^2) obtained by MD simulations and NMR relaxation for the amide NH bonds and Cα-RMSF extracted from the MD simulations.

	NMR S^2	MD simulation S^2	RMSF (nm)
TEM-1	0.90 ± 0.05	0.87 ± 0.05	0.07 ± 0.03
PSE-4	0.87 ± 0.05	0.86 ± 0.05	0.08 ± 0.04
cTEM-2m	N.D.	0.87 ± 0.05	0.09 ± 0.04
cTEM-17m	0.89 ± 0.05	0.87 ± 0.05	0.09 ± 0.05
cTEM-19m	N.D.	0.86 ± 0.05	0.10 ± 0.04

N.D.: not determined.

Supplementary Table S4. Cα-RMSD between the average structure from each 2 µs simulation and the crystal structure used for that simulation: TEM-1 (PDB 1XPB), PSE-4 (PDB 1G68), cTEM-2m (PDB 4MEZ), cTEM-17m (PDB 4ID4) and cTEM-19m (PDB 4R4S).

	Repetition 1	Repetition 2	Repetition 3	Average
PSE-4	1.21	1.11	1.40	1.24 ± 0.12
TEM-1	1.18	0.84	0.82	0.95 ± 0.20
cTEM-2m	1.14	1.03	1.18	1.12 ± 0.06
cTEM-17m	1.67	0.915	1.01	1.20 ± 0.34
cTEM-19m	1.69	1.624	1.52	1.61 ± 0.07

Supplementary Table S5. Per residue S^2 extracted from MD simulations for TEM-1, PSE-4, cTEM-2m, cTEM-17m and cTEM-19m compared to the NMR-determined S^2 for TEM-1, PSE-4 and cTEM-17m.

See EXCEL document

Supplementary Table S6. Per residue Cα-RMSF extracted from triplicate MD simulations of 2 µs each for TEM-1, PSE-4, cTEM-2m, cTEM-17m and cTEM-19m.

See EXCEL document

Supplementary Table S7. Differences in measured R_2 (1/ τcp) at fast (τcp = 0.625 ms) and slow (τcp = 10 ms) pulsing rates (ΔR_2 values ≥ $7 s^{-1}$), derived from TROSY-based ^{15}N-CPMG relaxation dispersion experiments, and unassigned residues (NA) for the chimeras cTEM-2m and cTEM-19m.

See EXCEL document

Supplementary Table S8. Comparison of the dynamism of the active-site walls using the S2 (ps-ns), Cα RMSF (ns-µs) and kex (µs-ms) on the different timescales.

	TEM-1			PSE-4			cTEM-2m			cTEM-17m			cTEM-19m		
	S^2	RMSF (nm)	k_{ex} (s^{-1})	S^2	RMSF (nm)	k_{ex} (s^{-1})	S^2	RMSF (nm)	k_{ex} (s^{-1})	S^2	RMSF (nm)	k_{ex} (s^{-1})	S^2	RMSF (nm)	k_{ex} (s^{-1})
**Protein average**	0.87 ± 0.05	0.07 ± 0.02	-	0.86 ± 0.05	0.08 ± 0.04	-	0.87 ± 0.05	0.09 ± 0.04	-	0.87 ± 0.05	0.09 ± 0.05	-	0.86 ± 0.05	0.10 ± 0.04	-
**S70 (69-73)**	0.91 ± 0.01	0.05 ± 0.01	-	0.90 ± 0.01	0.06 ± 0.01	-	0.89 ± 0.01	0.06 ± 0.01	940 ± 250 (69)	0.90 ± 0.01	0.06 ± 0.02	480 ± 110 (69, 72)	0.89 ± 0.01	0.06 ± 0.01	670 ± 140 (66, 72, 73)
**Y105 (103-106)**	0.86 ± 0.01	0.08 ± 0.02	-	0.85 ± 0.01	0.10 ± 0.03	-	0.85 ± 0.01	**0.14 ± 0.05**	-	0.86 ± 0.01	0.08 ± 0.01	300 ± 90 (105, 106)	0.85 ± 0.01	0.11 ± 0.04	1,220 ± 230 (105)
**SDN (129-132)**	0.89 ± 0.01	0.06 ± 0.02	-	0.87 ± 0.01	0.06 ± 0.01	-	0.88 ± 0.01	0.09 ± 0.02	480 ± 210 (130, 131)	0.88 ± 0.01	0.07 ± 0.01	780 ± 270 (128, 129)	0.87 ± 0.02	0.07 ± 0.02	980 ± 200 (128, 129, 130, 131)
**Ω-loop (162-179)**	0.85 ± 0.01	0.07 ± 0.01	-	0.85 ± 0.02	0.09 ± 0.02	2,020 ± 810 (173)	0.85 ± 0.02	0.10 ± 0.03	-	0.85 ± 0.03	**0.14 ± 0.09**	590 ± 130 (166, 170)	0.82 ± 0.03	**0.17 ± 0.09**	330 ± 100 (166, 170) ; Gly172: 2,110 ± 840
**Hinge leading in Ω-loop (155-160)**	**0.79 ± 0.02**	**0.13 ± 0.01**	-	**0.80 ± 0.03**	**0.14 ± 0.07**	-	**0.81 ± 0.03**	**0.12 ± 0.02**	-	**0.80 ± 0.03**	**0.12 ± 0.04**	-	**0.79 ± 0.03**	**0.15 ± 0.02**	-
**Tip of the Ω-loop (171-178)**	**0.79 ± 0.01**	**0.10 ± 0.01**	-	**0.80 ± 0.03**	**0.13 ± 0.02**	2,020 ± 810 (173)	**0.78 ± 0.04**	**0.13 ± 0.03**	-	**0.80 ± 0.04**	**0.21 ± 0.15**	-	**0.78 ± 0.03**	**0.23 ± 0.12**	-
**214-218**	**0.80 ± 0.04**	**0.15 ± 0.05**	-	0.85 ± 0.01	0.07 ± 0.02	-	**0.76 ± 0.04**	**0.25 ± 0.10**	1,320 ± 870 (212,	**0.77 ± 0.02**	**0.24 ± 0.08**	-	**0.78 ± 0.03**	**0.17 ± 0.04**	1,130 ± 420 (213, 217)
**connector (214-225)**	**0.81 ± 0.04**	**0.14 ± 0.05**	1,390 ± 370 (225)	0.85 ± 0.01	0.07 ± 0.02	-	**0.78 ± 0.04**	**0.21 ± 0.06**	1,340 ± 780 (212, 217, 221)	**0.78 ± 0.02**	**0.21 ± 0.07**	1,100 ± 440 (225)	**0.78 ± 0.03**	**0.17 ± 0.04**	820 ± 210 (213, 217, 225)
**234-244**	0.88 ± 0.01	0.06 ± 0.01	650 ± 30 (241)	0.89 ± 0.01	0.06 ± 0.01	-	0.88 ± 0.01	0.07 ± 0.01	610 ± 220 (238, 241, 243, 245, 246)	0.88 ± 0.01	0.07 ± 0.02	-	0.86 ± 0.02	0.09 ± 0.01	-
**β-strand 3 (230-237)**	0.88 ± 0.01	0.06 ± 0.01	-	0.90 ± 0.01	0.05 ± 0.01	-	0.89 ± 0.01	0.07 ± 0.01	-	0.89 ± 0.01	0.07 ± 0.02	910 ± 210 (231, 232, 233)	0.88 ± 0.01	0.06 ± 0.01	800 ± 250 (230, 231, 233)
**Turn β-strand 3/β-strand 4 (238-242)**	0.88 ± 0.01	0.07 ± 0.01	650 ± 30 (241)	0.88 ± 0.01	0.06 ± 0.01	-	0.88 ± 0.01	0.08 ± 0.01	600 ± 270 (238, 241, 243)	0.87 ± 0.01	0.08 ± 0.03	-	0.84 ± 0.02	0.12 ± 0.02	-
**H1 (26-41)**	0.88 ± 0.01	0.08 ± 0.01	-	0.85 ± 0.01	0.10 ± 0.01	-	0.88 ± 0.01	0.09 ± 0.01	470 ± 210 (38)	0.87 ± 0.01	0.09 ± 0.01	450 ± 60 (29, 32, 37, 38, 39, 41)	0.87 ± 0.01	0.10 ± 0.02	290 ± 50 (28, 29, 32, 38, 41)
**H11 (272-288)**	0.88 ± 0.01	0.10 ± 0.04	1,190 ± 340 (287)	0.86 ± 0.02	0.09 ± 0.02	-	0.87 ± 0.02	0.11 ± 0.02	800 ± 140 (276-277)	0.87 ± 0.01	0.13 ± 0.04	440 ± 50 (277, 278, 279, 281, 284, 287)	0.87 ± 0.01	0.12 ± 0.03	840 ± 60 (275, 276, 277, 278, 279, 280, 284, 287)

k_{ex} : residues used for the fitting are indicated between bracket

Supplementary Table S9. Kinetic parameters for hydrolysis of the cephalosporins cephalothin (CF), cefazolin (CZ) and cefotaxime (CTX) **and penicillins** benzylpenicillin (BZ) and carbenicillin (CB) by the β-lactamases TEM-1 and PSE-4, the chimeras cTEM-2m, cTEM-17m and cTEM-19m and the deconvoluted mutants (cTEM-17m context = cTEM-18m(M68L), cTEM-18m(M69T); TEM-1 context = TEM-1(M68L), TEM-1(M69T)).

	Variant	k_{cat} (s^{-1})	K_M (μM)	k_{cat}/K_M ($M^{-1}s^{-1}$)
CF	TEM-1[a]	84 ± 12	180 ± 28	4.3×10^5
	cTEM-17m[a]	120 ± 8	470 ± 80	2.4×10^5
	TEM-1(M68L)	31 ± 7	100 ± 30	3.0×10^5
	cTEM-18m(M68L)	34 ± 5	80 ± 38	5.0×10^5
	TEM-1(M69T)	7 ± 4	130 ± 11	5.2×10^4
	cTEM-18m(M69T)	1.4 ± 0.3	12 ± 3	1.2×10^5
	cTEM-2m	2.8 ± 0.8	46 ± 19	6.1×10^4
	cTEM-19m	6 ± 0.5	68 ± 14	8.8×10^5
	PSE-4[a]	0.80 ± 0.01	64 ± 34	1.3×10^4
CZ	TEM-1[a]	55 ± 8	130 ± 9	4.2×10^5
	cTEM-17m[a]	75 ± 21	600 ± 90	1.2×10^5
	TEM-1(M68L)	59 ± 21	500 ± 178	1.2×10^5
	cTEM-18m(M68L)	23 ± 5	170 ± 43	1.4×10^5
	TEM-1(M69T)	9 ± 2	300 ± 121	3.0×10^4
	cTEM-18m(M69T)	4 ± 0.6	83 ± 18	5.0×10^4
	cTEM-2m	3.3 ± 0.1	75 ± 1	4.4×10^4
	cTEM-19m	6 ± 0.3	120 ± 5	5.0×10^4
	PSE-4[a]	1.9 ± 0.3	140 ± 39	1.3×10^4
CTX	TEM-1[a]	0.74 ± 0.1	840 ± 160	8.8×10^2
	cTEM-17m[a]	0.14 ± 0.04	260 ± 100	5.6×10^2
	TEM-1(M68L)	0.06 ± 0.02	170 ± 72	3.6×10^2
	cTEM-18m(M68L)	0.07 ± 0.03	260 ± 73	2.5×10^2
	TEM-1(M69T)	0.03 ± 0.02	280 ± 151	1.1×10^2
	cTEM-18m(M69T)	0.06 ± 0.02	430 ± 78	1.4×10^2
	cTEM-2m	0.02 ± 0.01	240 ± 40	8.2×10^1
	cTEM-19m	0.008 ± 0.002	110 ± 37	7.0×10^1
	PSE-4[a]	0.03 ± 0.01	200 ± 30	1.5×10^2
BZ	TEM-1[a]	450 ± 100	20 ± 3	2.3×10^7
	cTEM-17m[a]	480 ± 46	28 ± 8	1.7×10^7
	TEM-1(M68L)	310 ± 19	11 ± 3	2.8×10^7
	cTEM-18m(M68L)	400 ± 50	31 ± 17	1.3×10^7
	TEM-1(M69T)	120 ± 3	7 ± 1	1.7×10^7
	cTEM-18m(M69T)	20 ± 5	6 ± 4	3.3×10^6
	cTEM-2m	83 ± 2	7 ± 1	1.2×10^7
	cTEM-19m*	230 ± 19*	< 1*	-
	PSE-4[a]	630 ± 110	16 ± 2	3.9×10^7
CB	TEM-1[a]	92 ± 4	49 ± 12	1.9×10^6
	cTEM-17m[a]	37 ± 2	22 ± 4	1.7×10^6
	TEM-1(M68L)	51 ± 3	28 ± 5	1.8×10^6
	cTEM-18m(M68L)	65 ± 13	37 ± 14	1.8×10^6
	TEM-1(M69T)	31 ± 3	12 ± 5	2.6×10^6
	cTEM-18m(M69T)	10 ± 5	26 ± 21	3.9×10^6
	cTEM-2m	24 ± 1	16 ± 3	1.5×10^6
	cTEM-19m	25 ± 6	27 ± 10	9.3×10^5
	PSE-4[a]	500 ± 92	64 ± 5	7.8×10^6

[a] Data from Clouthier *et al.* [2]

*k_{cat} (apparent) and estimated K_M due to saturation at a substrate concentration ≥ 5 μM.

Supplementary Table S10. Crystal contacts for each of the β-lactamases under study.

See EXCEL document

Residue	68	69	150	153	154	155	158	159	165	168	172	173	174	175	177	182	184	186	188
TEM-1	M	M	A	H	N	M	H	V	W	E	A	I	P	N	E	M	A	M	T
cTEM-2m	L	T																	
cTEM-17m	M	M																	
cTEM-19m	L	T	D	R	Q	I	K	E	I	D	G	K	L	G	L	T	K	I	S
PSE-4	L	T																	

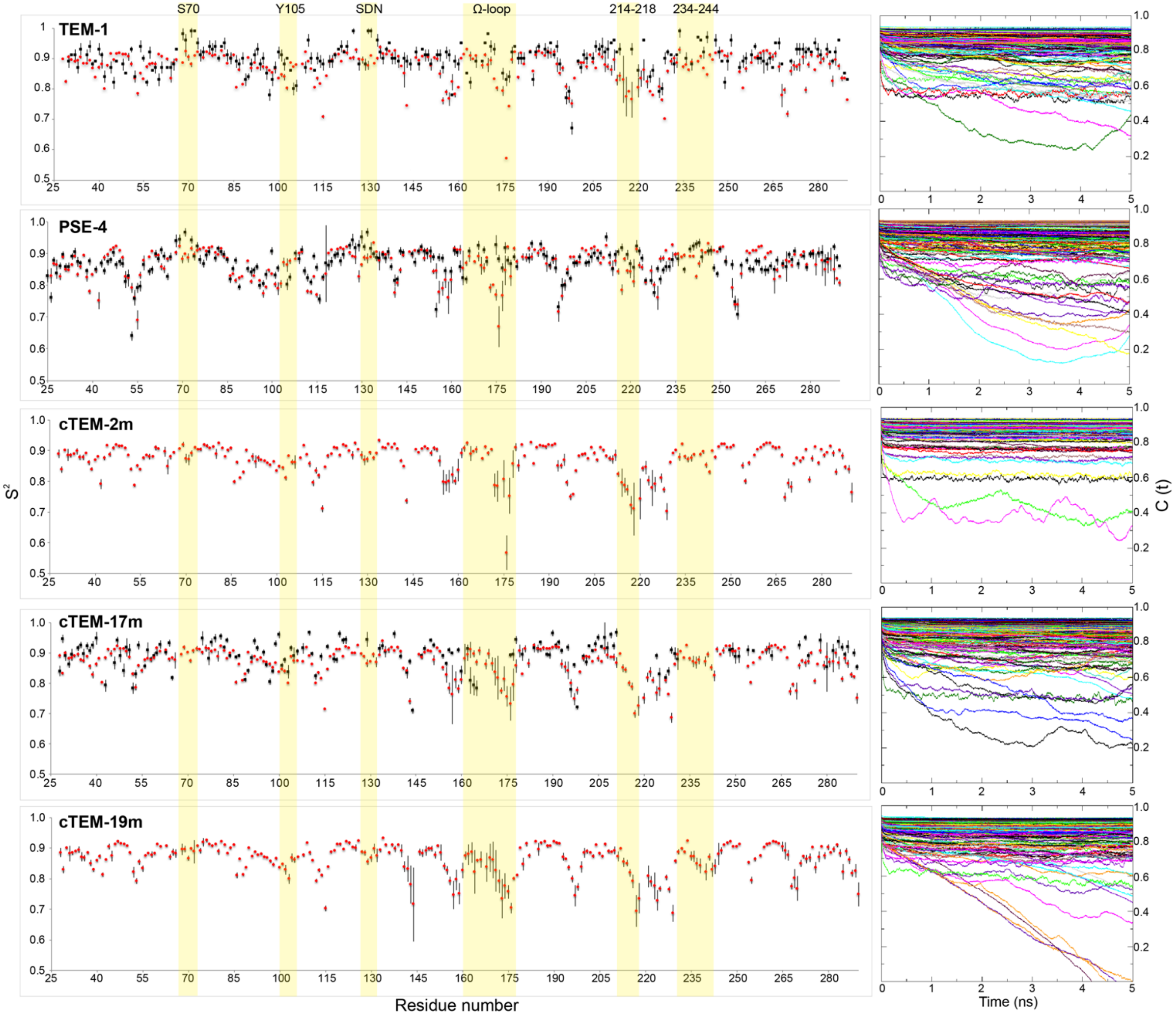

Supplementary Figure S1. Sequence relation in the exchanged regions of the chimeras (residues 66-73 and 150-190) to the parental TEM-1 and PSE-4 class A β-lactamases and generalized squared order parameters (S^2) along the protein sequence for the amide NH bonds. MD-derived (red) and NMR relaxation (black) S^2 are compared for TEM-1 and PSE-4 and the chimera cTEM-17m. MD-derived S^2 (red) are shown for cTEM-2m and cTEM-19m. The mean MD-NMR S^2 difference (or Δ) was the highest for Asp176 (Δ = 0.22 in TEM-1, 0.21 in PSE-4 and 0.16 in cTEM-17m), Asp115 (Δ = 0.17 in TEM-1; no value in cTEM-17m), Gly143 (Δ = 0.16 in TEM-1 and 0.13 in cTEM-17m) and Trp229 (Δ = 0.21 in TEM-1 and 0.22 in cTEM-17m). Examples of the autocorrelation functions for all residues for one among the 100 analyzed 10 ns segments is shown on the right. Error bars are the S^2 difference between all 10 ns segments of the simulations, for each residue.

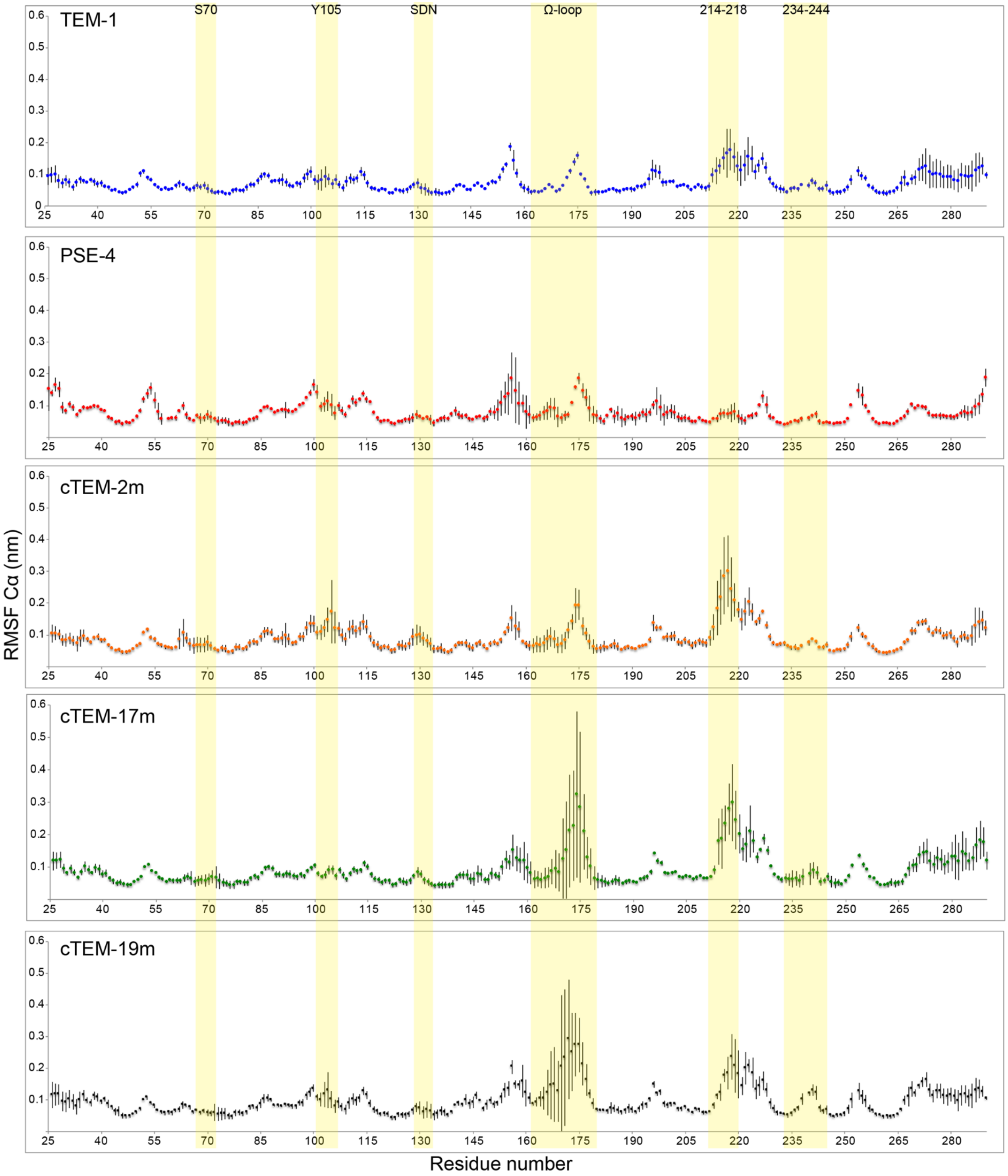

Supplementary Figure S2. Cα RMSF derived from triplicate 2 µs MD simulations for the parental class A β-lactamases TEM-1 and PSE-4 and the chimeras cTEM-2m, cTEM-17m and cTEM-19m. Error bars are the RMSF difference between replicas for each residue.

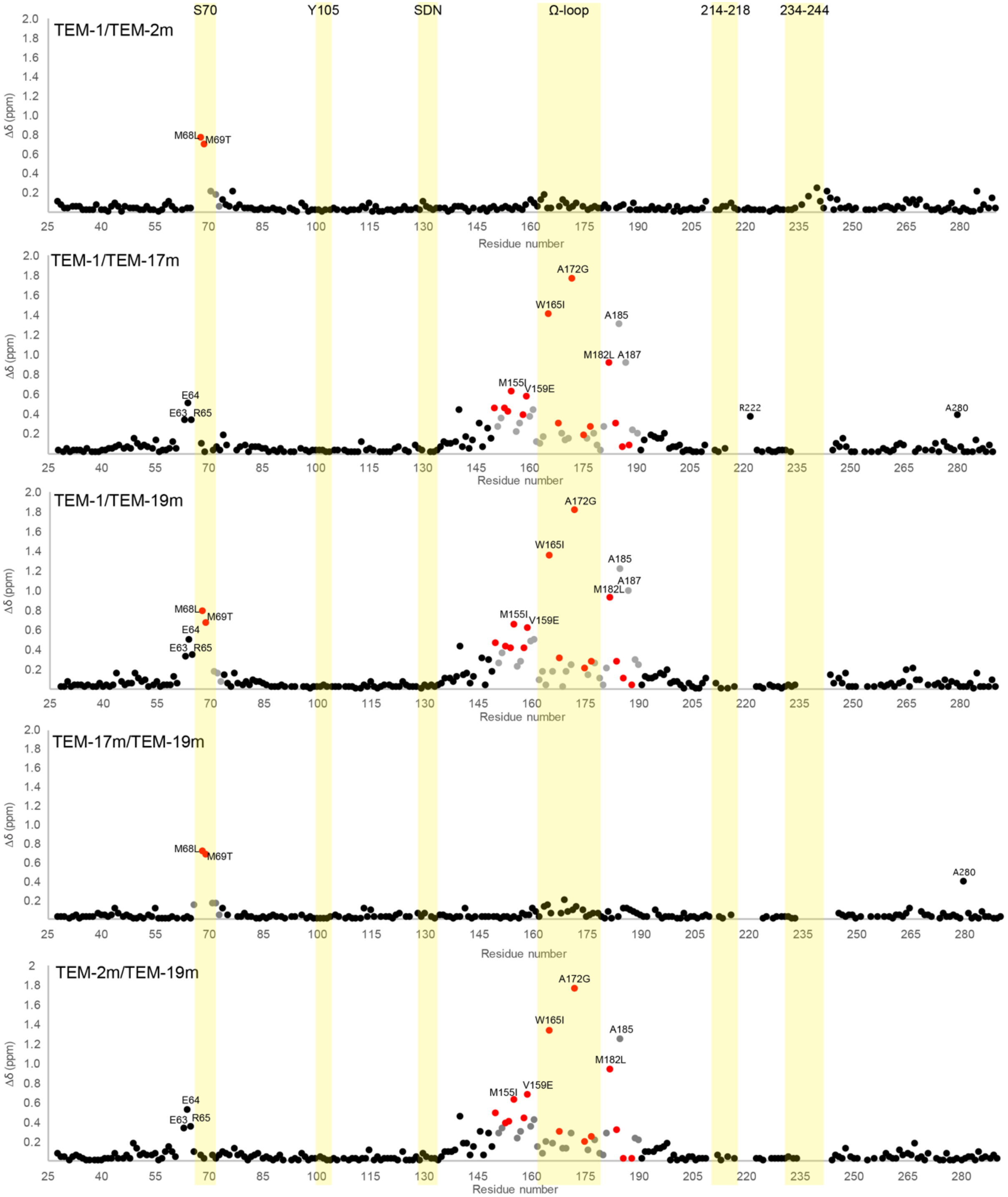

Supplementary Figure S3. Effect of the recombination of regions 66-73 and 150-190 on the backbone chemical shifts. Comparison of the chimeras cTEM-2m, cTEM-17m and cTEM-19m to the parental TEM-1 class A β-lactamases and comparisons of cTEM-2m and cTEM-17m to cTEM-19m. Exchanged residues relative to TEM-1 sequence are colored red while residues in the exchanged regions (66 to 73 and 150 to 190) are colored grey.

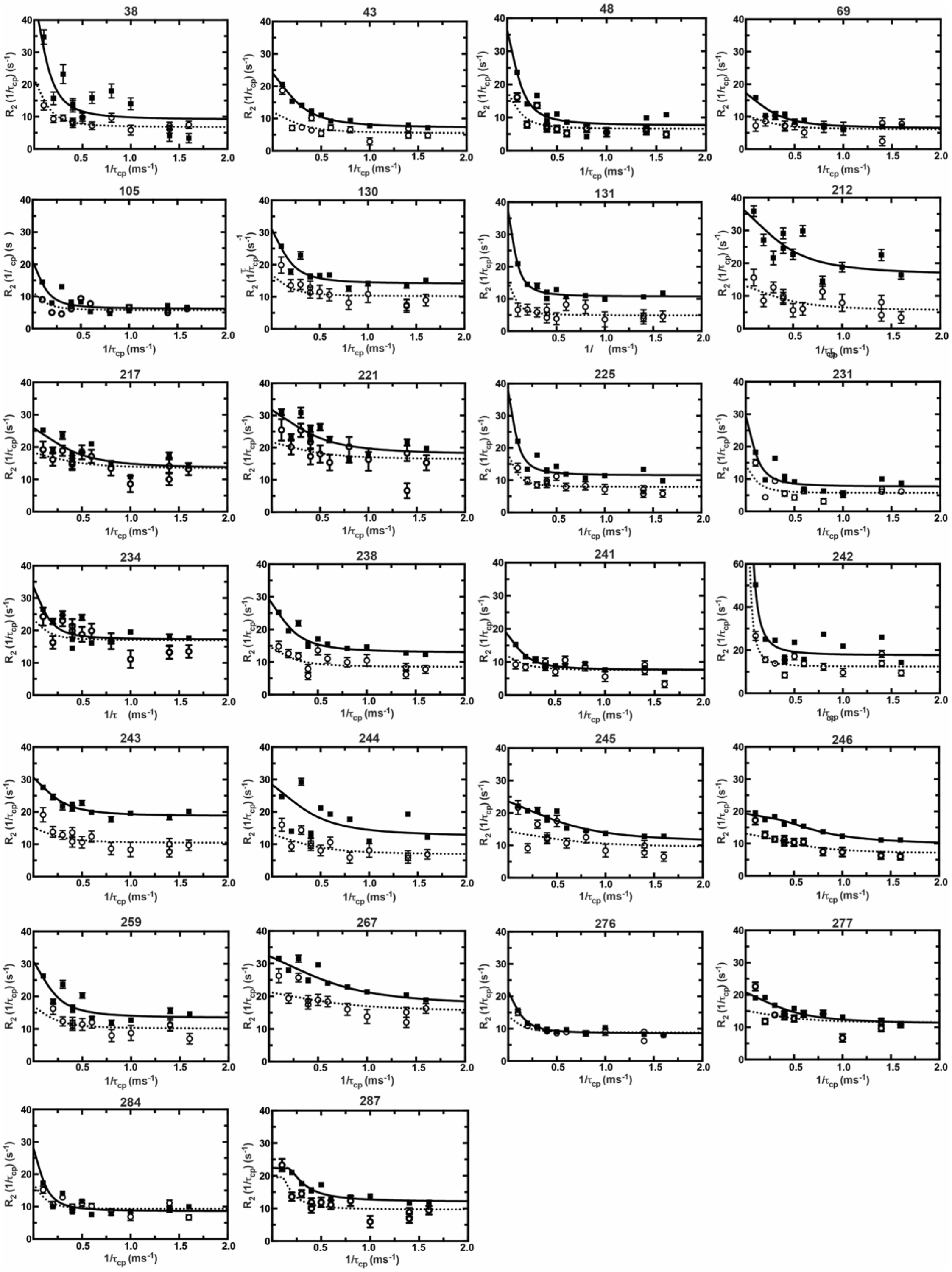

Supplementary Figure S4a. ^{15}N-CPMG relaxation dispersion curves for cTEM-2m at 800 MHz (full line) and 500 MHz (dashed line). The residue number appears above each plot.

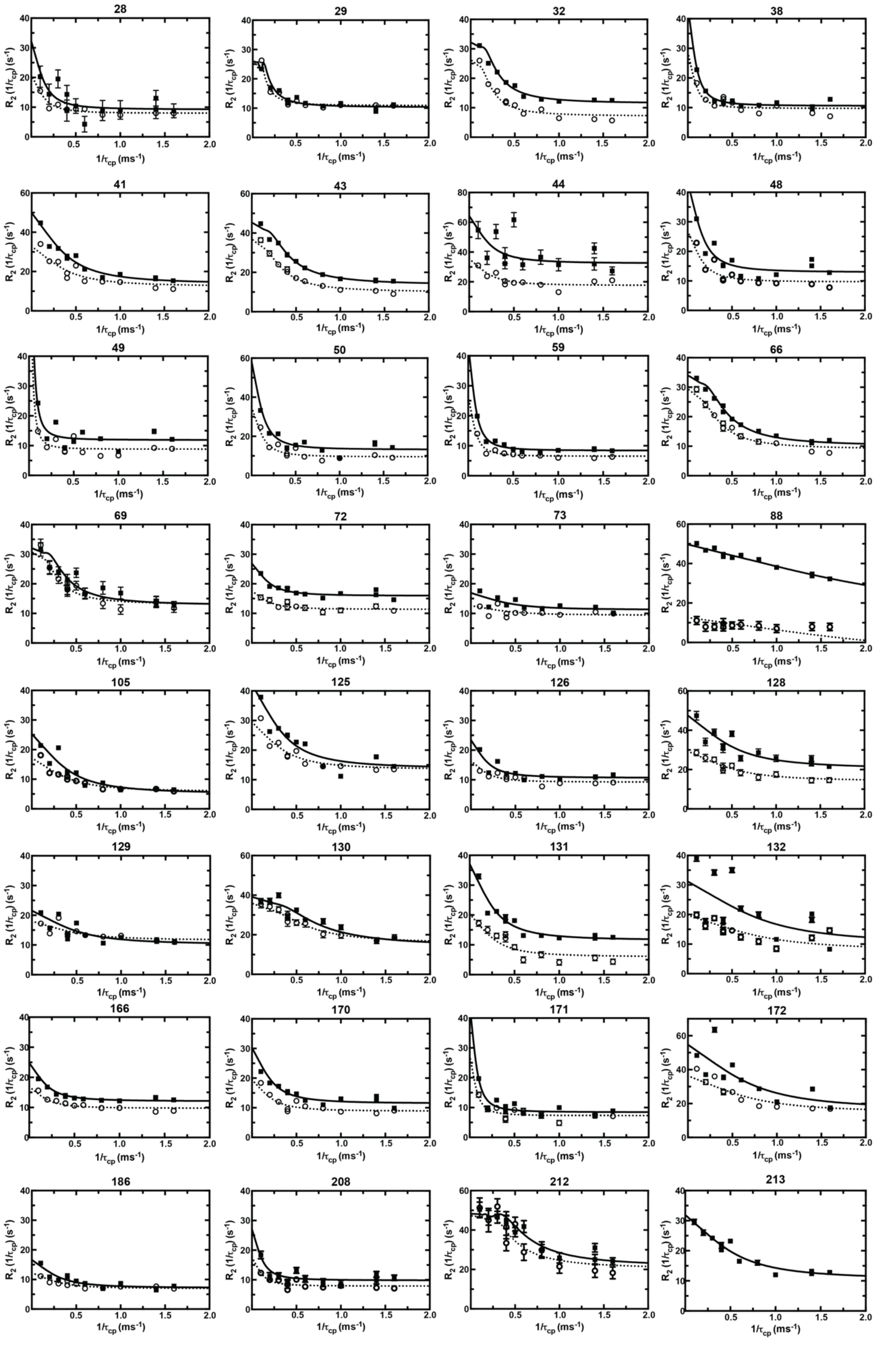
28
29
32
38
41
43
44
48
49
50
59
66
69
72
73
88
105
125
126
128
129
130
131
132
166
170
171
172
186
208
212
213
$R_2\ (1/\tau_{cp})\ (s^{-1})$
$1/\tau_{cp}\ (ms^{-1})$

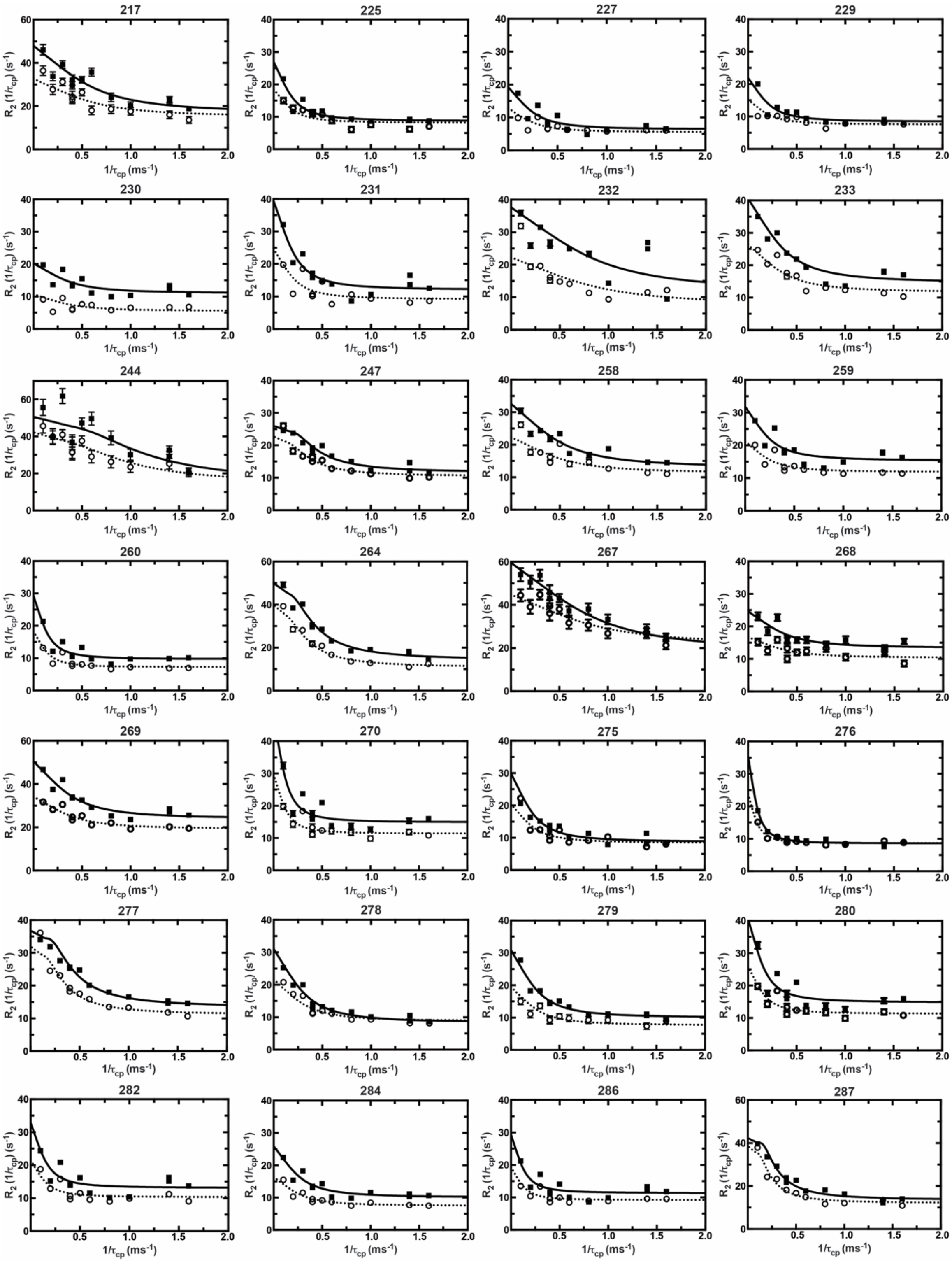

Supplementary Figure S4b. ^{15}N-CPMG relaxation dispersion curves for cTEM-19m at 800 MHz (full line) and 600 MHz (dashed line). The residue number appears above each plot.

SUPPLEMENTARY MATERIALS AND METHODS

Materials. All DNA-modifying enzymes were purchased from New England Biolabs (Mississauga, ON) or Bio-Rad (Mississauga, ON). Nitrocefin was purchased from Calbiochem (Mississauga, ON). Kanamycin (Kan), isopropyl-1-thio-β-D-galactopyranoside (IPTG), components for growth media and purification materials were from Bioshop Canada (Burlington, ON). 99% Ammonium chloride ($^{15}NH_4Cl$) and 99% deuterium oxide (D_2O) were purchased from Cambridge Isotope Laboratories (Tewksbury, MA). 99% D-glucose-^{13}C, benzylpenicillin (BZ), carbenicillin (CB), cephalotin (CF), cefazolin (CZ), cefotaxime (CTX), DEAE-Sepharose Fast Flow, Tris-HCl, PEG 4000, HEPES, ammonium sulfate and $MgCl_2$ used for protein crystallization were purchased from Sigma-Aldrich (Oakville, ON). Tazobactam was purchased from Molekula (Dorset, UK).

Mutagenesis and subcloning. Subcloning of the chimera cTEM-19m into pET-24 with the OmpA signal sequence was performed as previously described [2]. Oligonucleotide primers used for mutagenesis of TEM-1 and cTEM-19m were synthesized by Alpha DNA (Montréal, QC). To generate the mutants, the QuickChange site-directed mutagenesis method [3] was used with the I-Proof High Fidelity DNA polymerase (Bio-Rad, Mississauga, ON). The PCR product was *Dpn*I-digested for 1 hour at 37°C to eliminate the starting DNA template. The digested product was butanol-precipitated and transformed into *E. coli* XL1 Blue. Cells were propagated for 1h in Luria-Bertani broth (LB) then plated on LB-agar containing 50 µg/mL Kan. Colonies were picked and propagated overnight. DNA extraction was performed for confirmation of DNA sequence of the entire coding region and for retransformation into *E. coli* BL21(DE3).

β-Lactamase expression and purification. Protein expression for enzymatic assays and crystallisation was performed as previously reported [2,4-6]. Briefly, cells were propagated in auto-inducing ZYP-5052 medium [7] (50 µg/mL Kan) with agitation at 37°C for two hours, then overnight at 22°C. Induced cells were sedimented by centrifugation (5,000 × *g*, 30 min, 4°C) and resuspended in 10 mM Tris-HCl buffer pH 7.0. Lysis was performed using a Constant Systems cell disrupter (Northants, UK). Cellular debris were sedimented by centrifugation (20,000 × *g*, 30 min, 4°C), the supernatant was filtered through a 0.2 µm filter and the protein was purified. The chromatography was undertaken at 4°C using an ÄKTA FPLC (GE Healthcare). Following sample application, the DEAE-Sepharose Fast Flow column (1.6 cm × 30 cm) was washed with 3 column volumes (CV) of 10 mM Tris-Cl pH 7.0. A linear gradient to 200 mM Tris-Cl pH 7.0 was achieved over 4 CV, and the column was further washed over 3 CV. Fractions containing β-lactamase were identified first using a qualitative nitrocefin hydrolysis essay and then by SDS-polyacrylamide gel electrophoresis (15% (w/v) polyacrylamide gel) with Coomassie Brilliant Blue staining. Analysis of the purity was performed with Image Lab 5.0 (Bio-Rad, Hercules, CA). Enzyme concentration was determined by Bradford assay using the Bio-Rad protein assay solution (Bio-Rad, Hercules, CA), with bovine serum albumin as the standard. For protein crystallization assay, a second purification step was added. Fractions containing ≥ 75% β-lactamase following the first purification step were concentrated to a volume of 1.5 mL using a 10,000 MWCO Amicon concentrator (Millipore, Billerica, MA) and applied to a Superose 12 column (1.6 cm × 55 cm) that had been pre-equilibrated with 50 mM Tris-HCl pH 7.0. Elution was performed at a flow rate of 1 mL/min. Fractions containing β-lactamase were identified by SDS-polyacrylamide gel electrophoresis (15% (w/v) polyacrylamide gel) with zinc-imidazole staining [8]. Purity and concentration were determined as above. Fractions containing β-lactamase with a purity of ≥ 95% were concentrated to 25 mg/mL (0.8 mM). For NMR assays, uniformly [^{15}N]- and [^{2}H,

^{15}N]-labeled cTEM-19m and cTEM-2m samples were produced by overexpression in modified M9 minimal media containing $^{15}NH_4Cl$ and deuterium oxide D_2O (99%) [2,4-6,9]. For [^2H, ^{15}N]-labeled samples, cells were progressively acclimatised to deuterium oxide using M9 media with 30, 60, 75, 85 and 99% D_2O. Purification was performed as described for protein crystallization assay. Samples were dialyzed against distilled, deionized water overnight at 4°C. Purity of the final samples was verified by SDS-PAGE stained by the zinc-imidazole method. Typical yields were 25 mg/L of > 98% pure protein.

cTEM-19m crystallization and data collection. cTEM-19m was concentrated to 25 mg/mL in 50 mM Tris-Cl (pH 7.0) in the presence and absence of 4 mM tazobactam (5-fold molar excess relative to the protein concentration). Crystals were grown at 22°C in hanging drops prepared by mixing 1 µL of the protein solution and 1 µL of the reservoir solution (100 mM Tris-Cl (pH 8.0), 26% PEG 4000, and 0.25 M $MgCl_2$). Two single crystals, one grown in the presence of tazobactam and the other in its absence, were isolated and flash-frozen in liquid nitrogen. Diffraction data were collected from each crystal at the Canadian Macromolecular Crystallography Facility Beamline 08ID-1 (Canadian Light Source, Saskatoon, SK). Although the classical β-lactamase inhibitor tazobactam (turnover rate of 1.4 s^{-1}) [10] was included during crystallization for cTEM-19m, no corresponding electron density was observed in 4R4S.

cTEM-2m crystallization and data collection. cTEM-2m was concentrated to 25 mg/mL in 50 mM Tris-Cl (pH 7.0). Crystals were grown at 22°C in hanging drops prepared by mixing 1 µL of the protein solution and 1 µL of the reservoir solution, which contained 100 mM HEPES (pH 7.5) and 1.5 M ammonium sulfate. A rod-shaped crystal was removed from a crystal cluster, transferred to a cryoprotectant solution containing 10% (v/v) glycerol, 100 mM HEPES (pH 7.5) and 1.5 M ammonium sulfate, and flash-cooled in a nitrogen gas cold-stream. Diffraction data were collected at 100K as 0.5° oscillation images, with a Rigaku MicroMax 007 HF X-ray generator and a Rigaku Saturn 944 HG CCD detector.

Crystallization data processing and refinement. The collected diffraction images were indexed, integrated, and scaled either with HKL2000 [11] or the *xia2* package [12]. Initial phases were calculated by molecular replacement with the program PHASER [13], with a previously determined TEM-1 structure (PDB code: 1ZG4) [14] as a search model. The structure models were further improved through iterative rounds of manual model building with COOT [15] and automated refinement with PHENIX [16] and REFMAC5 [17]. Data collection and refinement statistics are summarized in **Supplementary Table 2**. The refined structures of cTEM-19m (PDB IDs 4R4R and 4R4S) and cTEM-2m (PDB ID: 4MEZ) have been deposited to the Protein Data Bank.

Active-site Volume Estimation. The active-site cavity volume of TEM-1 (PDB 1XPB), PSE-4 (PDB 1G68), cTEM-2m (PDB 4MEZ), cTEM-17m (PDB 4ID4) and cTEM-19m (PDB 4R4S) was estimated using 3V: Voss Volume Voxelator [18]. The estimation was made with a small sphere of 1.5Å radius and a large sphere of 8 Å radius.

Molecular dynamics simulations. Crystal structures with the best ratio of resolution and completeness of the sequence were chosen for the two parents (TEM-1: 1XPB, 1.9 Å resolution and PSE-4: 1G68, 1.95 Å resolution) and the three variants (cTEM-2m: 4MEZ, 2.05 Å resolution, cTEM-17m: 4ID4, 1.05 Å resolution and cTEM-19m: 4R4S, 1.1 Å resolution). These structures were used as a starting point for all molecular dynamics (MD) simulations. Missing side-chains in 4MEZ (LYS32, LYS55, LYS111, ASP115, LYS146, LYS1192 and ARG277), 4ID4 (LYS32, LYS55, LYS111, LYS146, GLN154, LYS158, LYS173, ASP254 and LYS256) and 4R4S (LYS 111, GLN 154, LYS 158, LYS215 and LYS256) were

reconstructed using the rotamer explorer in the Structure Preparation plugin of MOE [19]. All following steps were executed using GROMACS 5.0.1. The protonation and orientation of histidines was verified using the high-resolution crystal structure of TEM-1 variant M182T (1M40), MolProbity [20] and PROPKA [21,22]. For each system, crystallographic water molecules were conserved in a truncated dodecahedron periodic water box of SPC/E water molecules with a minimum distance of 10 Å between the box boundary and the protein. The total number of water molecules and net charge for the different systems were: 1XPB: 12,812, -7; 1G68: 12,475, -5; 4MEZ: 9,952, -7; 4ID4: 10,177, -4; 4R4R: 10,703, -4. Each system was neutralized by adding the respective number of sodium counterions. The total size of the systems, in atoms, was: 1XPB: 42,507; 1G68: 41,501; 4MEZ: 33,926, 4ID4: 34,619; 4R4R: 36,196.

For energy minimization and MD simulations, GROMACS 5.0.1 was used with the AMBER99SB-ILDN force field [23,24]. The LINear Constraint Solver (LINCS) algorithm was applied to all bonds containing hydrogen atoms. Each 1 ps frame was saved in a compressed XTC trajectory and each 100 ps in a full precision TRR trajectory. The Nose-Hoover thermostat and the Parrinello-Rahman barostat were used to couple the system to a constant temperature of 304.65 K and a pressure of 1 bar. The electrostatic interactions were evaluated by the particle-mesh Ewald method, and Lennard-Jones interactions were evaluated using a 1.2 nm cutoff. Each system was energy minimized using the method of the Steepest Descent to a target F_{max} of no greater than 1,000 kJ mol^{-1} nm^{-1}. The time step was chosen to be 2 fs. Initial equilibration for conformation generation: All systems were subjected to 2 ns NVT ensemble to gradually heat up the system from 10 K to 304.65 K. Next, a 20 ns NPT ensemble was generated and three conformations with the highest, average and lowest root mean-square deviation (RMSD) were chosen as starting conformations for three individual replicates. A second equilibration for each replica was used to generate new and independent velocities: a 2 ns NVT ensemble was performed for each repetition to heat up the system and used as starting point for the 2 μs production run. This results in a total of three 2 μs simulations with 1 ps compressed and 100 ps full precision trajectories for each of the five proteins.

Analysis of protein structure and dynamics. Analysis was performed using GROMACS 5.0.1 on 2 μs for each MD simulation, unless otherwise stated. All protein figures were created using PyMOL [25] or MOE (Chemical Computing Group, Montréal, QC). The stability of the system was evaluated after 100 ns and 1,000 ns by calculating the RMSD of the backbone atoms between each frame and the initial conformation. In addition, the radius of gyration was calculated for each replicate over 2 μs to ensure overall structural integrity. The root mean-square fluctuation (RMSF) was calculated based on the Cα atom for each residue and converted into B-factors after removal of the overall translational and rotational motion of the protein by superimposition onto a common reference frame.

S^2 order parameters were calculated applying the model-free approach of Lipari and Szabo [26,27]. After removal of translational and rotational motion for each 2 μs run, the trajectory was split into 10 ns segments. For every second segment, the autocorrelation function was calculated for each N-H internuclear vector. The final S^2 value was obtained by averaging all 10 ns segments, thus representing the S^2 value over 2 μs.

Solution NMR experiments. All NMR experiments were performed as previously reported [4,5]. Briefly, [^{15}N] and [^{2}H,^{15}N]-Labeled samples were concentrated to 0.6 mM using a 10,000 MWCO Amicon concentrator and buffer exchanged to 3 mM imidazole, 0.01% NaN_3, and 10% D_2O, pH 6.7. The final pH was verified using a pH meter with a microprobe. The temperature was calibrated to 31.5°C with a standard methanol sample [28] on Agilent 800 MHz (18.8T –

cTEM-2m and cTEM-19m), Agilent 600 MHz (14.1T, cTEM-19m) and Agilent 500 MHz (11.7T, cTEM-2m) NMR spectrometers, each equipped with a triple-resonance probe and a pulse-field gradient. Microsecond-millisecond protein dynamics for cTEM-2m and cTEM-19m were characterized using ^{15}N TROSY relaxation-compensated Carr-Purcell-Meiboom-Gill (rcCPMG) experiments [29]. Transverse spin-relaxation data using the TROSY rcCPMG experiment were acquired in an interleaved fashion with interpulse delays, τ_{cp}, of 0.0, 0.625, 0.714 (× 2), 1.0, 1.25, 1.67, 2.0, 2.50 (× 2), 3.33, 5.0, and 10.0 ms during the nitrogen relaxation period for a constant, total relaxation time of 20 ms. All heteronuclear NMR data were processed using NMRPipe [30], in-house scripts and further analyzed with Sparky (T. D. Goddard and D. G. Kneller, SPARKY 3, University of California, San Francisco). Previously acquired and analysed NMR backbone assignments of cTEM-2m (BMRB 26586) and cTEM-19m (BMRB 26590) were used to identify the N-H resonances [5]. Conformational exchange was considered significant when the difference in measured R_2 ($\Delta R_2\,(1/\tau_{cp})$) at fast ($\tau_{cp}$ = 0.625 ms) and slow (τ_{cp} = 10 ms) pulsing rates on the 18.8T data set was greater than 7 s^{-1} in relaxation dispersion profiles. This threshold was selected based on relaxation dispersion error estimation, to eliminate false positives resulting from a poor fit and to be consistent with the previously reported dynamics of TEM-1, PSE-4 and cTEM-17m [4]. Global residue fits and model analyses were performed using the 800 MHz (cTEM-2m and cTEM-19m) in combination with the 500 MHz (cTEM-2m) or 600 MHz (cTEM-19m) relaxation dispersion data and fitting them using GraphPad Prism 5 to the full single-quantum ^{15}N-CPMG equation [31]. Residues were selected for global fitting as a region, first according to structural proximity and second according to statistically overlapping k_{ex}.

Enzyme kinetics. K_M and k_{cat} parameters were determined at room temperature in 50 mM sodium phosphate buffer, pH 7.0. The following extinction coefficients (and concentration ranges) were used: $\Delta\varepsilon_{232\ nm}$ = 900 $M^{-1}cm^{-1}$ for BZ (5-240 μM) [32], $\Delta\varepsilon_{232\ nm}$ = 1,190 $M^{-1}cm^{-1}$ for CB (5-240 μM) [33], $\Delta\varepsilon_{262\ nm}$ = 7,960 $M^{-1}cm^{-1}$ for CF (5-250 μM) [32], $\Delta\varepsilon_{260\ nm}$ = 7,900 $M^{-1}cm^{-1}$ for CZ (5-250 μM) [34], and $\Delta\varepsilon_{264\ nm}$ = 7,250 $M^{-1}cm^{-1}$ for CTX (10-240 μM) [35]. Substrate hydrolysis was monitored according to initial steady-state velocities by using a Cary100 Bio UV-visible spectrophotometer (Agilent Technologies Canada, Montréal, QC) and quartz cuvettes with a path length of either 1 cm (CF, CZ, and CTX) or 10 cm (BZ and CB). Six substrate concentrations were used, generally flanking the K_M values for TEM-1. The enzyme concentrations were maintained within 5 to 30 nM. The kinetic parameters were determined from the hydrolysis rates calculated from the initial linear portion of the curve. Data fitting to the Henri Michaelis-Menten equation for the substrates CB and BZ was carried out with GraphPad Prism (GraphPad Software, San Diego, CA). For CF, CZ, and CTX a Lineweaver-Burk model (1/V *vs* 1/[S]) was used with Microsoft Excel due to inability to saturate the enzyme, a common issue with these substrates.

SUPPLEMENTARY REFERENCES

1 Zeil, C., Widmann, M., Fademrecht, S., Vogel, C. & Pleiss, J. Network Analysis of Sequence-Function Relationships and Exploration of Sequence Space of TEM β-Lactamases. *Antimicrobial Agents and Chemotherapy* **60**, 2709-2717, doi:10.1128/aac.02930-15 (2016).

2 Clouthier, C. M. *et al.* Chimeric beta-lactamases: global conservation of parental function and fast time-scale dynamics with increased slow motions. *PloS one* **7**, e52283, doi:10.1371/journal.pone.0052283 (2012).

3 Papworth C, B. J., Braman J, Wright DA. Site-directed mutagenesis in one day with >80% efficiency. *Strategies* **9**, 3-4 (1996).

4 Gobeil, S. M. *et al.* Maintenance of Native-like Protein Dynamics May Not Be Required for Engineering Functional Proteins. *Chemistry & biology*, doi:10.1016/j.chembiol.2014.07.016 (2014).

5 Gobeil, S. M., Gagné, D., Doucet, N. & Pelletier, J. N. N, C and H backbone resonance assignments of an artificially engineered TEM-1/PSE-4 class A beta-lactamase chimera and its deconvoluted mutant. *Biomol NMR Assign*, doi:10.1007/s12104-015-9645-8 (2015).

6 Morin, S., Clouthier, C. M., Gobeil, S., Pelletier, J. N. & Gagné, S. M. Backbone resonance assignments of an artificially engineered TEM-1/PSE-4 Class A beta-lactamase chimera. *Biomol NMR Assign* **4**, 127-130, doi:10.1007/s12104-010-9227-8 (2010).

7 Studier, F. W. Protein production by auto-induction in high density shaking cultures. *Protein Expr Purif* **41**, 207-234 (2005).

8 Simpson, R. J. Zinc/Imidazole Procedure for Visualization of Proteins in Gels by Negative Staining. *Cold Spring Harbor Protocols* **2007**, pdb.prot4701-pdb.prot4701, doi:10.1101/pdb.prot4701 (2007).

9 Morin, S., Levesque, R. C. & Gagné, S. M. 1H, 13C, and 15N backbone resonance assignments for PSE-4, a 29.5 kDa class A beta-lactamase from Pseudomonas aeruginosa. *Journal of biomolecular NMR* **36 Suppl 1**, 11, doi:10.1007/s10858-005-5343-7 (2006).

10 Swaren, P. *et al.* X-ray structure of the Asn276Asp variant of the Escherichia coli TEM-1 beta-lactamase: direct observation of electrostatic modulation in resistance to inactivation by clavulanic acid. *Biochemistry* **38**, 9570-9576, doi:10.1021/bi990758z (1999).

11 Otwinowski, Z. & Minor, W. Processing of X-ray diffraction data collected in oscillation mode. *Method Enzymol* **276**, 307-326 (1997).

12 Winter, G., Lobley, C. M. & Prince, S. M. Decision making in xia2. *Acta Crystallogr D Biol Crystallogr* **69**, 1260-1273, doi:10.1107/S0907444913015308 (2013).

13 McCoy, A. J. *et al.* Phaser crystallographic software. *Journal of applied crystallography* **40**, 658-674, doi:10.1107/S0021889807021206 (2007).

14 Stec, B., Holtz, K. M., Wojciechowski, C. L. & Kantrowitz, E. R. Structure of the wild-type TEM-1 beta-lactamase at 1.55 A and the mutant enzyme Ser70Ala at 2.1 A suggest the mode of noncovalent catalysis for the mutant enzyme. *Acta Crystallogr D Biol Crystallogr* **61**, 1072-1079, doi:10.1107/S0907444905014356 (2005).

15 Emsley, P. & Cowtan, K. Coot: model-building tools for molecular graphics. *Acta Crystallogr D Biol Crystallogr* **60**, 2126-2132, doi:10.1107/S0907444904019158 (2004).

16 Adams, P. D. *et al.* PHENIX: a comprehensive Python-based system for macromolecular structure solution. *Acta Crystallogr D Biol Crystallogr* **66**, 213-221, doi:10.1107/S0907444909052925 (2010).

17 Vagin, A. A. *et al.* REFMAC5 dictionary: organization of prior chemical knowledge and guidelines for its use. *Acta Crystallogr D* **60**, 2184-2195, doi:Doi 10.1107/S0907444904023510 (2004).
18 Voss, N. R. & Gerstein, M. 3V: cavity, channel and cleft volume calculator and extractor. *Nucleic Acids Research* **38**, W555-W562, doi:10.1093/nar/gkq395 (2010).
19 2016 (Chemical Computing Group Inc.).
20 Chen, V. B. *et al.* MolProbity: all-atom structure validation for macromolecular crystallography. *Acta Crystallogr D Biol Crystallogr* **66**, 12-21, doi:10.1107/S0907444909042073 (2010).
21 Sondergaard, C. R., Olsson, M. H., Rostkowski, M. & Jensen, J. H. Improved Treatment of Ligands and Coupling Effects in Empirical Calculation and Rationalization of pKa Values. *J Chem Theory Comput* **7**, 2284-2295, doi:10.1021/ct200133y (2011).
22 Olsson, M. H., Sondergaard, C. R., Rostkowski, M. & Jensen, J. H. PROPKA3: Consistent Treatment of Internal and Surface Residues in Empirical pKa Predictions. *J Chem Theory Comput* **7**, 525-537, doi:10.1021/ct100578z (2011).
23 Lindorff-Larsen, K. *et al.* Improved side-chain torsion potentials for the Amber ff99SB protein force field. *Proteins* **78**, 1950-1958, doi:10.1002/prot.22711 (2010).
24 Abraham, M. J. *et al.* GROMACS: High performance molecular simulations through multi-level parallelism from laptops to supercomputers. *SoftwareX* **1-2**, 19-25, doi:10.1016/j.softx.2015.06.001 (2015).
25 The PyMOL Molecular Graphics System v. Version 1.8.
26 Lipari, G. & Szabo, A. Model-Free Approach to the Interpretation of Nuclear Magnetic-Resonance Relaxation in Macromolecules .1. Theory and Range of Validity. *Journal of the American Chemical Society* **104**, 4546-4559, doi:DOI 10.1021/ja00381a009 (1982).
27 Lipari, G. S., A. Model-free approach to the interpretation of nuclear magnetic resonance relaxation in macromolecules. 2. Analysis of experimental results. *Journal of the American Chemical Society* **104**, 4559-4570, doi:10.1021/ja00381a010 (1982).
28 John Cavanagh, W. J. F., Arthur G. Palmer III, Mark Rance and Nicholas J. Skelton. *Protein NMR Spectroscopy: Principles and Practice*. Second Edition edn, (2007).
29 Loria, J. P., Rance, M. & Palmer, A. G., 3rd. A TROSY CPMG sequence for characterizing chemical exchange in large proteins. *Journal of biomolecular NMR* **15**, 151-155 (1999).
30 Delaglio, F. *et al.* NMRPipe: a multidimensional spectral processing system based on UNIX pipes. *Journal of biomolecular NMR* **6**, 277-293 (1995).
31 Manley, G. & Loria, J. P. NMR insights into protein allostery. *Archives of Biochemistry and Biophysics* **519**, 223-231, doi:10.1016/j.abb.2011.10.023 (2012).
32 Bouthors, A. T. *et al.* Role of residues 104, 164, 166, 238 and 240 in the substrate profile of PER-1 beta-lactamase hydrolysing third-generation cephalosporins. *Biochemical Journal* **330**, 1443-1449 (1998).
33 Lim, D. *et al.* Insights into the molecular basis for the carbenicillinase activity of PSE-4 beta-lactamase from crystallographic and kinetic studies. *Biochemistry* **40**, 395-402 (2001).
34 Tribuddharat, C., Moore, R. A., Baker, P. & Woods, D. E. Burkholderia pseudomallei class A beta-lactamase mutations that confer selective resistance against ceftazidime or clavulanic acid inhibition. *Antimicrob Agents Ch* **47**, 2082-2087 (2003).
35 Cantu, C. & Palzkill, T. The role of residue 238 of TEM-1 beta-lactamase in the hydrolysis of extended-spectrum antibiotics. *Journal of Biological Chemistry* **273**, 26603-26609 (1998).
